# The *Cryptococcus neoformans* Titan cell is an inducible and regulated morphotype underlying pathogenesis

**DOI:** 10.1371/journal.ppat.1006978

**Published:** 2018-05-18

**Authors:** Ivy M. Dambuza, Thomas Drake, Ambre Chapuis, Xin Zhou, Joao Correia, Leanne Taylor-Smith, Nathalie LeGrave, Tim Rasmussen, Matthew C. Fisher, Tihana Bicanic, Thomas S. Harrison, Marcel Jaspars, Robin C. May, Gordon D. Brown, Raif Yuecel, Donna M. MacCallum, Elizabeth R. Ballou

**Affiliations:** 1 Medical Research Council Centre for Medical Mycology at the University of Aberdeen, Aberdeen Fungal Group, Institute of Medical Sciences, Foresterhill, Aberdeen, United Kingdom; 2 Institute of Microbiology and Infection, School of Biosciences, University of Birmingham, Edgbaston, United Kingdom; 3 Marine Biodiscovery Centre, Department of Chemistry, University of Aberdeen, Aberdeen, United Kingdom; 4 Francis Crick Institute, London, United Kingdom; 5 Institut für Biochemie, Universität Würzburg, Wurzburg, Germany; 6 Dpt. Infectious Disease Epidemiology, Imperial College London, London, United Kingdom; 7 Institute of Infection and Immunity, St George’s University of London, London, United Kingdom; Carnegie Mellon University, UNITED STATES

## Abstract

Fungal cells change shape in response to environmental stimuli, and these morphogenic transitions drive pathogenesis and niche adaptation. For example, dimorphic fungi switch between yeast and hyphae in response to changing temperature. The basidiomycete *Cryptococcus neoformans* undergoes an unusual morphogenetic transition in the host lung from haploid yeast to large, highly polyploid cells termed Titan cells. Titan cells influence fungal interaction with host cells, including through increased drug resistance, altered cell size, and altered Pathogen Associated Molecular Pattern exposure. Despite the important role these cells play in pathogenesis, understanding the environmental stimuli that drive the morphological transition, and the molecular mechanisms underlying their unique biology, has been hampered by the lack of a reproducible *in vitro* induction system. Here we demonstrate reproducible *in vitro* Titan cell induction in response to environmental stimuli consistent with the host lung. *In vitro* Titan cells exhibit all the properties of *in vivo* generated Titan cells, the current gold standard, including altered capsule, cell wall, size, high mother cell ploidy, and aneuploid progeny. We identify the bacterial peptidoglycan subunit Muramyl Dipeptide as a serum compound associated with shift in cell size and ploidy, and demonstrate the capacity of bronchial lavage fluid and bacterial co-culture to induce Titanisation. Additionally, we demonstrate the capacity of our assay to identify established (cAMP/PKA) and previously undescribed (*USV101*) regulators of Titanisation *in vitro*. Finally, we investigate the Titanisation capacity of clinical isolates and their impact on disease outcome. Together, these findings provide new insight into the environmental stimuli and molecular mechanisms underlying the yeast-to-Titan transition and establish an essential *in vitro* model for the future characterization of this important morphotype.

## Author summary

Changes in cell shape underlie fungal pathogenesis by allowing immune evasion and dissemination. *Aspergillus* and *Candida albicans* hyphae drive tissue penetration. *Histoplasma capsulatum* and *C*. *albicans* yeast growth allows evasion and dissemination. As major virulence determinants, morphogenic transitions are extensively studied in animal models and *in vitro*. The pathogenic fungus *Cryptococcus neoformans* is a budding yeast that, in the host lung, switches to an unusual morphotype termed the Titan cell. Titans are large, highly polyploid, have altered cell wall and capsule, and produce haploid daughters. Their size prevents engulfment by phagocytes, yet they are linked to dissemination and altered immune response. Despite their important influence on disease, replicating the yeast-to-Titan switch *in vitro* has proved challenging. Here we show that Titans are induced by host-relevant stimuli, including serum and bronchio-alveolar lavage fluid. We identify a bacterial cell wall component as a relevant inducing compound and predict an *in vivo* Titan defect for a clinical isolate. Genes regulating *in vivo* Titanisation also influence *in vitro* formation. Titanisation is a conserved morphogenic switch across the *C*. *neoformans* species complex. Together, we show that Titan cells are a regulated morphotype analogous to the yeast-to-hyphal transition and establish new ways to study Titans outside the host lung.

## Introduction

Fungi change shape in response to environmental stimuli. These morphogenic transitions drive pathogenesis and allow fungi to occupy different environmental niches. Dimorphic fungi undergo a yeast-to-hyphal transition in response to changing temperature, while the pleomorphic gut resident fungus *Candida albicans* integrates diverse signals depending on its local environment [1, 2]. The basidiomycete *Cryptococcus neoformans* undergoes an unusual transition in the host lung from haploid yeast-phase growth to apolar expansion and endo-reduplication, producing large, highly polyploid cells termed Titan cells[3, 4]. While there is growing evidence of the important role Titan cells play in disease [5–8], understanding the mechanisms underlying the yeast-to-Titan transition remains challenging due to the lack of an *in vitro* model.

*C*. *neoformans* is an environmental human pathogen that causes cryptococcal meningitis when inhaled yeast and spores disseminate to the central nervous system and brain. The fungus infects an estimated 1 million people worldwide each year and is responsible for between 140,000 and 600,000 deaths, primarily in sub-Saharan Africa [9–11]. Although the majority of patients are immunocompromised, a growing number of infections are seen in immuno-competent individuals [12–14]. Long term azole therapy is associated with relapse due to drug resistance and the emergence of hetero-resistance [14–16]. *C*. *neoformans* grows preferentially as an encapsulated budding yeast under physiologically relevant conditions and during culture in standard microbial media, and the vast majority of research has focused on the yeast form. However, there are early clinical reports of Titan cells, in which large encapsulated yeast were isolated from the lung and brain of infected patients [17, 18]. In both cases, cell size was dependent on growth condition, shifting from >40 μm in patient samples to <20 μm during in vitro culture and back to >40 μm in murine infection. Cruickshank et al. also report distinct capsule and cell wall structure of enlarged cells[18]. Despite this clear morphological transition, both early reports concluded that the patient samples represented atypical isolates.

However, far from being unusual outliers, it is now clear that Titan cells represent a unique aspect of cryptococcal biology. Recent work in mouse models of infection have demonstrated that Titan cells comprise 20% of fungal cells in the lung and are associated with dissemination to the brain and a non-protective immune response [5, 7, 8, 19]. Titanisation requires the activity of the Gα protein Gpa1 and the G-protein coupled receptor Gpr5, as well as the mating pheromone receptor Ste3**a**, likely targeting the cAMP/PKA pathway [20]. Transcription factors that influence cAMP-regulated capsule and melanin also influence Titanisation [20–22]. However, the environmental triggers of Titanisation remain unknown, and reports of *in vitro* Titanisation have not led to a robust *in vitro* protocol for their generation [4, 20, 23, 24].

The analysis of Titan cells recovered from infected mice has led to the identification of four defining features: Titans are larger than 10 μm, are polyploid (typically 4-8C, although higher ploidies have been reported), have a tightly compacted capsule, and have a dramatically thicker cell wall [6, 8, 18]. Here we report a simple and robust protocol for the *in vitro* generation of cells matching this definition. We validate the capacity of our protocol to identify genes required for Titanisation, and predict the capacity of clinical isolates to form Titans. Finally, we identify environmentally relevant ligands that trigger the yeast-to-Titan transition and begin to dissect the underlying molecular mechanisms that drive this novel virulence mechanism.

## Results

### Large polyploid cells can be induced *in vitro*

**Fig 1:**
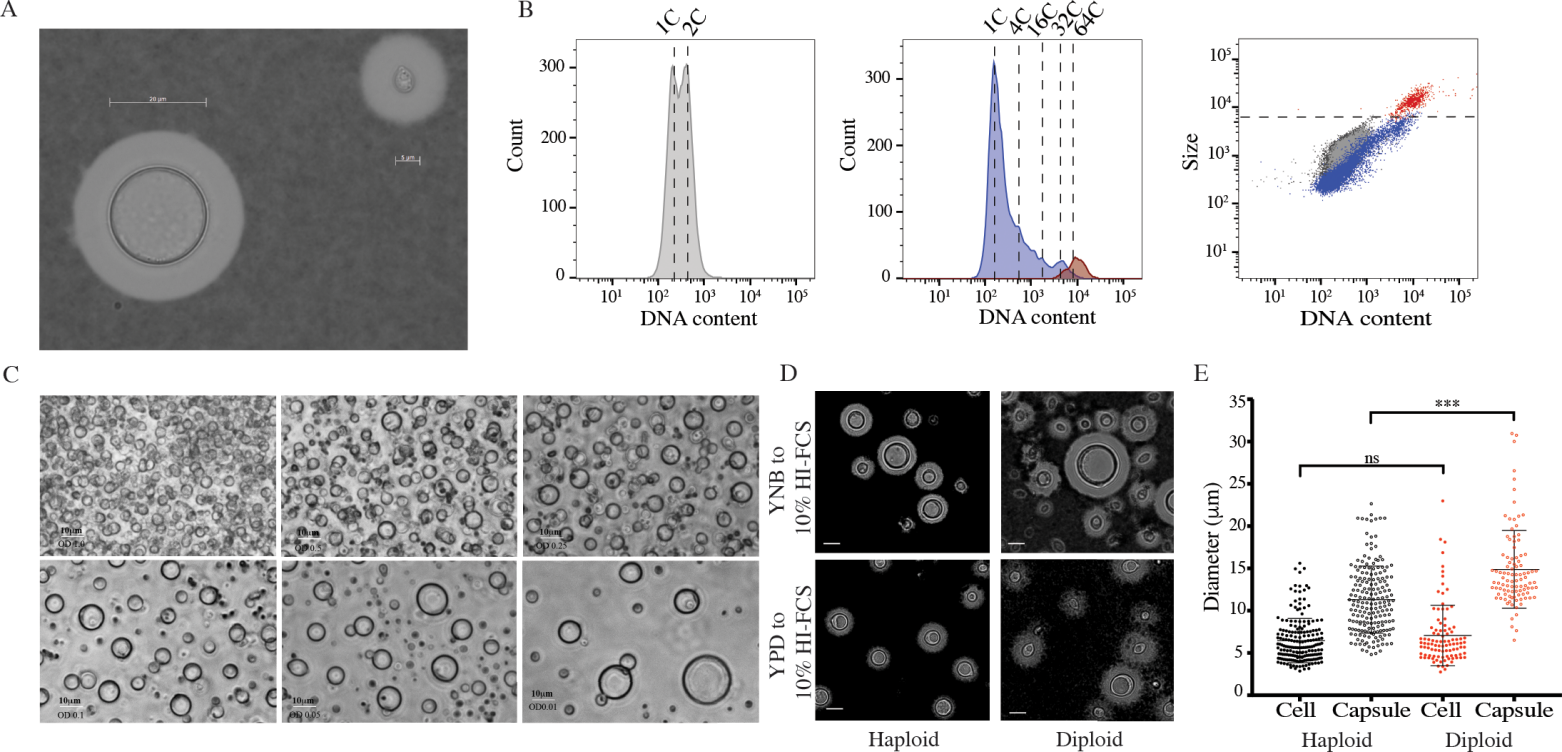
Titan cells can be induced *in vitro* and have all the properties of *in vivo* induced cells. H99 haploid cells pre-grown in YNB and incubated in 10%HI-FCS at 37°C, 5%CO_2_ for 3 days. Resulting colonies were A) counterstained with India ink to reveal capsule and B) fixed and stained for DNA content relative to haploid and diploid controls. Left panel shows YNB grown haploid control. Middle panel shows total induced population (blue). In addition, induced cells were passaged through an 11 μm filter to enrich for large cells (red). Right panel shows all three populations. C) H99 haploid cells were pre-grown in YNB and incubated in 10%HI-FCS at the indicated OD_600_ at 37°C, 5%CO_2_ for 3 days. Scale bar = 10 μm. D,E) H99 haploid and KN994B7#16 diploid cells pre-grown in either YNB or YPD were inoculated into 10%HI-FCS at OD_600_ = 0.001, (37°C, 5%CO_2_, 3 days) and then counterstained with India ink and analysed. Representative micrographs (D) and diameters for >200 cells excluding or including capsule (E) are shown. Significance was assessed using Mann Whitney U (***p<0.0001).

While investigating the impact of nutrient starvation on virulence factor production, we observed that when *C*. *neoformans* cells grown in Yeast Nitrogen Base (YNB) with 2% glucose were transferred to 10% HI-FCS at 5% CO_2_, 37°C, large cells (up to 50 μm) formed after several days (Fig 1A, 3 days; S1A Fig, 7 days). These cells expressed a compact capsule that was readily distinguished from the more typical yeast capsule by India ink staining (Fig 1A). Similar effects were observed for cells grown in 10% native FCS or heat inactivated (HI)-FCS but not for culture-matched cells transferred to 1xPBS. Given the reported capacity of *C*. *neoformans* to form polyploid Titan cells, these large cells were examined for DNA content. Induced cells were passed through an 11 μm filter to enrich for large cells. Both fractions were collected, fixed and stained for DNA content (Fig 1B, S1B Fig gating strategy). While un-induced cells showed distinct 1C and 2C peaks representing progression of haploid yeast through the cell cycle, induced cells additionally showed discrete peaks consistent with populations of higher ploidy cells. The filtered population was enriched for large cells with increased DNA content (Fig 1B), consistent with these large cells being polyploid Titan cells.

Titan cells observed *in vivo* typically comprise up to 20% of the total cell population, and lower inocula are associated with an increase in the proportion of Titan cells [20]. We likewise observed that a minority of cells fell into the >10 μm category and that the percentage and overall cell size increased at decreasing inoculum concentration (Fig 1C). While large cells were readily observed at OD_600_ = 0.25, there was an increase in the frequency and size of larger cells at lower optical densities (OD_600_ 0.05, 0.01). Optimization revealed that larger than average cells (10–12 μm) could be observed after 24 hr, but that cells approaching 15 μm were readily observed after 72 hr. Therefore, all subsequent characterizations were performed using an overnight culture of YNB+Glucose grown cells inoculated into 1xPBS + 10% HI-FCS at OD_600_ = 0.001 and incubated for 72 hr at 37°C, 5% CO_2_. The induced phenotype was reproducible across labs and users (University of Aberdeen (TD, ERB), University of Birmingham (ERB, LTS, XZ), Clemson University (LK)).

### *In vitro* generated large cells are Titan cells

Where yeast cells typically range in size from 5–7 μm, Titan cells have been defined as being >10 μm or >15 μm, and some definitions have included capsule (>30 μm) [20, 23]. When we measured the cell body diameter of H99 induced cells, we observed a size range spanning 3–15 μm (Fig 1D and 1E). Cells >10 μm represented 15.72 ± 4.46% of the population. We also observed that cells with a diploid base ploidy tended to produce a higher proportion of cells >15 μm (Fig 1D and 1E). Cell size and ploidy are proportional, and we tested the impact of base ploidy on induced cell size. When cell body alone was considered, there was a shift in the size and frequency of cells >10 μm between haploid (H99) and diploid (KN994B7#16) base ploidy, although this did not reach significance (Fig 1E; p = 0.0785). When capsule was taken into account, the difference in size became highly significant (Fig 1E; p<0.0001), suggesting that capsule size increases with base ploidy. Additionally, we observed that cell:capsule ratios were not uniform across the entire population as cell body size increased (S1C Fig): The cell:capsule ratio was significantly smaller for cells >10 μm than for cells <10 μm (H99: 1.425 vs. 1.696; p<0.0001).

**Fig 2:**
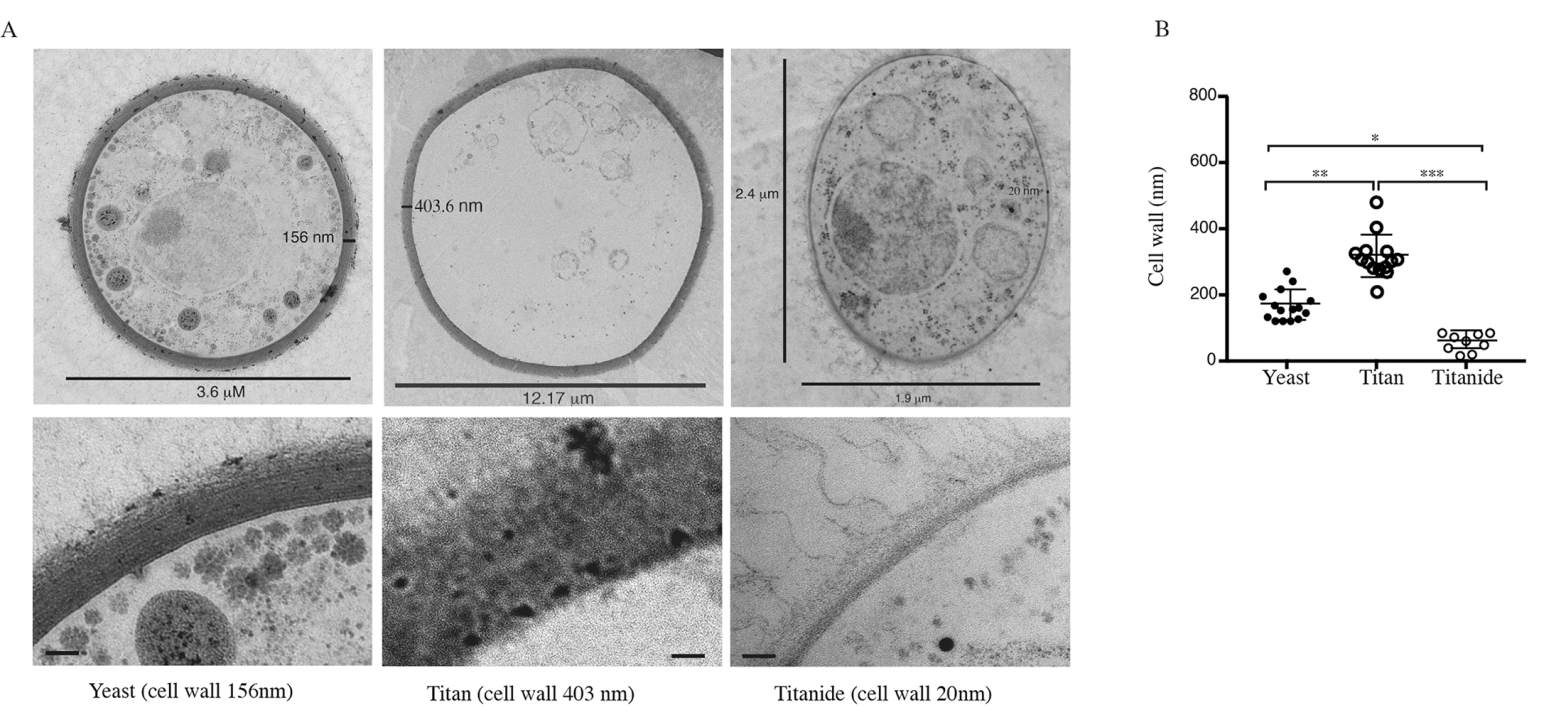
*In vitro* induced cells were analysed by TEM. Representative micrographs (A) and cell wall thickness for Yeast (n = 15), Titan (n = 14), and Titanide (n = 9) cells (B). Significance was assessed using Kruskal-Wallis and Dunn's multiple comparisons tests (* p = 0.027; ** p = 0.002; ***p<0.0001).

**Fig 3:**
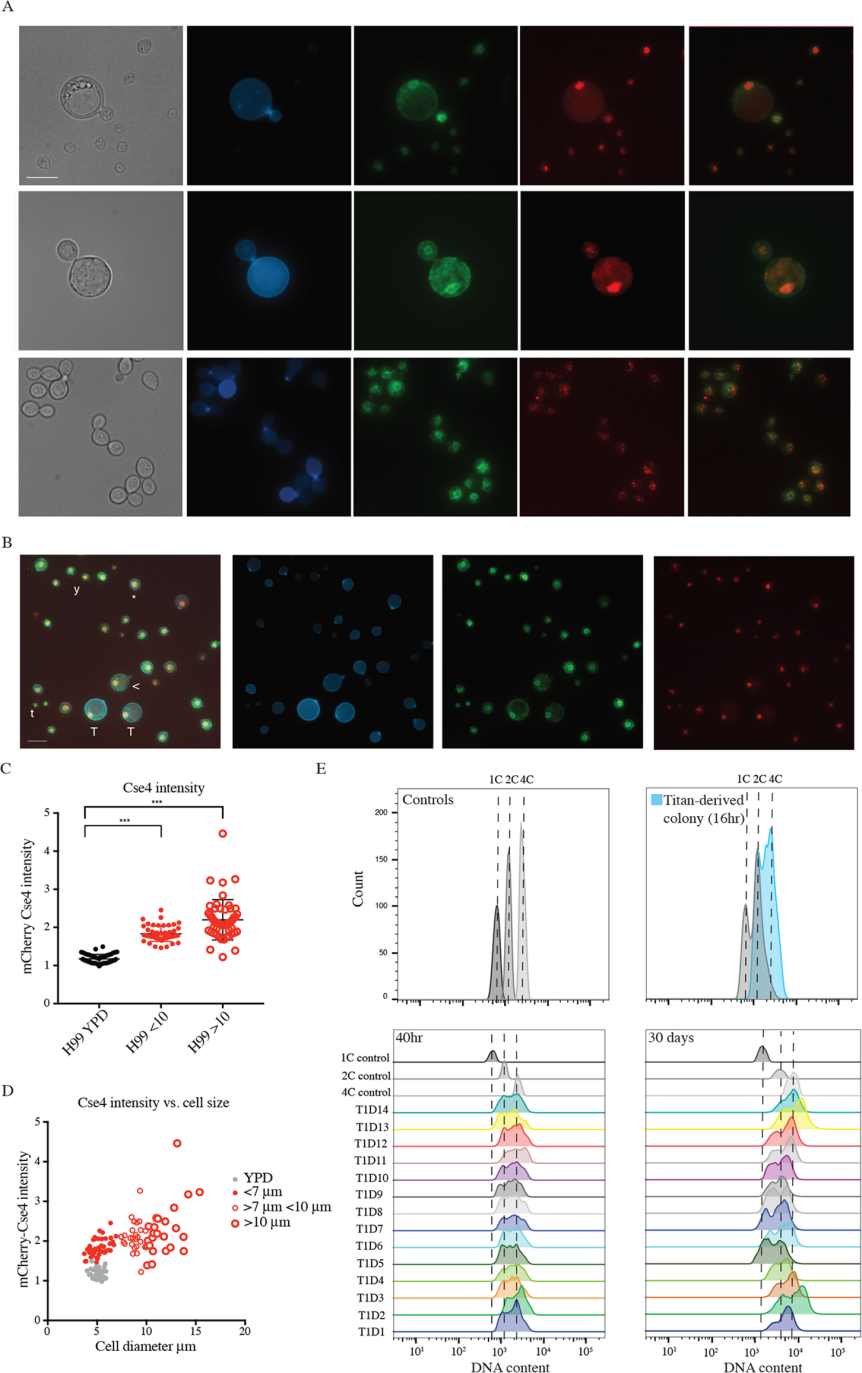
Daughter cells from *in vitro* Titans have altered ploidy relative to the haploid parent. A) Representative budding Titan cells (top panels) and yeast cells (bottom panel). CNV111 haploid cells encoding GFP-Ndc1 (membrane) and mCherry-Cse4 (kinetochore) were induced (top) or grown in YPD (bottom) and stained for chitin (CFW, 10 μg/ml). Scale bar = 10 μm. B) Induced CNV111 cells showing population heterogeneity (T,Titan; Y,Yeast; t, Titanide; (*), aneuploid; (<), less than 10 μm). Scale bar = 10 μm. C,D) CNV111 cells were quantified for mCherry-Cse4 foci and mother cell size (YPD n = 50, induced n = 100). E) Daughters arising from a single Titan mother were isolated and allowed to proliferate for 72 hr at 30°C on YPD agar prior to fixation and staining for DNA content (DAPI). Populations were analysed by flow cytometry. (Top) Control gates (1C, 2C, 4C) are shown aligned to representative overlays of ungated haploid control and Titan Daughter #5 (T1D5) populations. (Bottom) DNA content of 14 independent daughter colonies 48 hr after isolation (right) and again after 1 month incubation at RT on YPD agar.

Titan cells have been reported to have thicker cell walls than yeast cells and to contain a single large vacuole of unknown function. TEM analysis (Fig 2A and 2B) revealed that cells >10 μm had significantly thicker cell walls (314.7 ± 64.0 nm) than those <10 μm (167.3 ± 46.2 nm; p = 0.002). Large cells were mostly devoid of organelles. In rare instances, some cytoplasmic material could be observed along the cell cortex of large cells, consistent with the presence of a large vacuole (S1D Fig). A third population of small (2–4 μm) cells was also observed (Figs 2A, 2B, 3A and 3B). TEM revealed that these small, encapsulated cells resembled yeast in that they appeared metabolically active, with ribosomes, mitochondria, and nucleus readily visualized, and capsule observed extending from the cell wall (Fig 2A). However, where yeast and yeast daughters are round, these tended to be oval and had significantly thinner cell walls (56.14 ± 26.8 nm; p = 0.026) (Fig 2B). These cells appear to be distinct from the previously reported micro-cells, defined as <1 μm with thick cell walls [25]. Because of their association with Titan inducing conditions, we here term these cells Titanides in order to distinguish them from yeast and micro-cells.

Titan cells are uninucleate, highly polyploid, and produce haploid, aneuploid, or diploid daughters [6, 23]. To investigate these features in *in vitro* induced cells, we used the GFP-Ndc1 mCherry-Cse4 reporter strain CNV111 [26]. GFP-Ndc1 is targeted to the nuclear envelope and mCherry-Cse4 labels a proportion of kinetochores in non-dividing cells. In yeast phase cells, GFP-Ndc1 could be observed surrounding a cluster of mCherry-positive points. Under inducing conditions, large cells likewise contained a single nucleus and were capable of passing DNA to daughter cells (Fig 3A).

Haploid *C*. *neoformans* cells have 15 chromosomes [27]. The mCherry-Cse4 reporter has been used as a proxy for nuclear content, where individual points represent chromosomes [26]. In our hands, we never observed more than 9 distinguishable foci in log-phase haploid cultures, and most cells showed 4 foci arrayed within the nuclear membrane, consistent with previous reports [26] (Fig 3A). When Cse4 foci were quantified using z-stack images, un-induced cells grown in YPD had on average 3.96 ±1.363 foci (n = 200) (Fig 3A, S1E Fig). Growth in YNB did not significantly impact the number of resolvable foci (4.26 ±1.342 (n = 200); p = 0.048; S1E Fig). In YNB-FCS induced cells >10 μm it was not possible to fully resolve the densely-packed foci (Fig 3A and 3B). To further investigate, DNA content in Titan mother and budded cells was estimated based on fluorescence intensity (Fig 3A and 3B representative overlay image). We observed a statistically significant overall increase in nuclear fluorescence intensity in cells >10 μm compared to YPD grown cells (mean: 2.281 ± 0.66 vs. 1.172 ± 0.12; p<0.0001; max: 4.462 vs. 1.419; Fig 3C). However, in budding Titans, we observed daughters with resolvable foci and DNA content consistent with haploid cells (Fig 3A and 3C). These observations are consistent with reports that polyploid Titans divide DNA asymmetrically, producing haploid daughter cells. Based on these data, including size, capsule, cell wall, and ploidy, we suggest that YNB-serum induced large cells are in fact *bona fide* Titan cells.

### Titanized populations are highly heterogeneous

Having identified robust conditions capable of replicating *in vivo* Titan induction, we set out to more closely observe changes in DNA content following large cell induction. Induced populations such as those presented in Fig 3B were examined for mCherry-Cse4 intensity, including cells <10 μm, predicted to comprise a mix of yeast and Titan daughters. We observed an overall increase in fluorescence intensity relative to YPD grown yeast (Fig 3C, 1.94 ± 0.3, p<0.0001), suggesting aneuploidy in the population. These cells were also larger on average than YPD-grown cells (5.678 ± 0.74 vs. 5.071 ± 0.60; p = 0.0007, n>50, Fig 3D). Closer examination of this population showed it to be highly heterogeneous, with cell size ranging from 2 μm to 9.9 μm (Fig 3B), and individual cells <10 μm exhibited a wide range in size and relative fluorescence (Fig 3B and 3D). In some instances, cells <10 μm closely resembled cells >10 μm in terms of morphology and nuclear content (Fig 3B, compare T and **<**). In other instances, yeast sized cells displayed higher than normal relative mCherry-Cse4 fluorescence (Fig 3B, compare Y and *). We also observed cells much smaller than yeast size with mCherry Cse4 fluorescence typical of yeast (Fig 2B, compare Y and t). This heterogeneity is represented graphically in Fig 3D (n = 100 induced cells total). In general, nuclear content was proportional to cell size (Fig 3D).

To further study the impact of induction on cell ploidy, and to rule out condition-dependent artefactual changes in fluorescence, we analysed induced cells incubated on YPD by flow cytometry. Individual Titans (>10μm) were isolated by microdissection and allowed to proliferate on YPD agar at 30°C for 16 hr. The entire colony was picked and immediately fixed for analysis (Fig 3E top). In addition, individual daughters were dissected from Titan-derived colonies and further incubated on YPD agar at 30°C for 24 hr to form colonies. Fig 3E shows representative flow cytometry data measuring DNA content for fourteen daughter cells arising from a single Titan mother. At the time of dissection, these daughters were diploid or aneuploid relative to the H99 haploid parent (Fig 3E, lower right) and showed cell size consistent with diploid DNA content (S2A Fig). Daughters were incubated on YPD agar at 25°C for 1 month and then analysed again. While some daughters resolved back to haploidy, others were stable within this time scale (Fig 3E, lower left).

### Titan cells form in response to sequential signals and can produce further Titan cells

**Fig 4:**
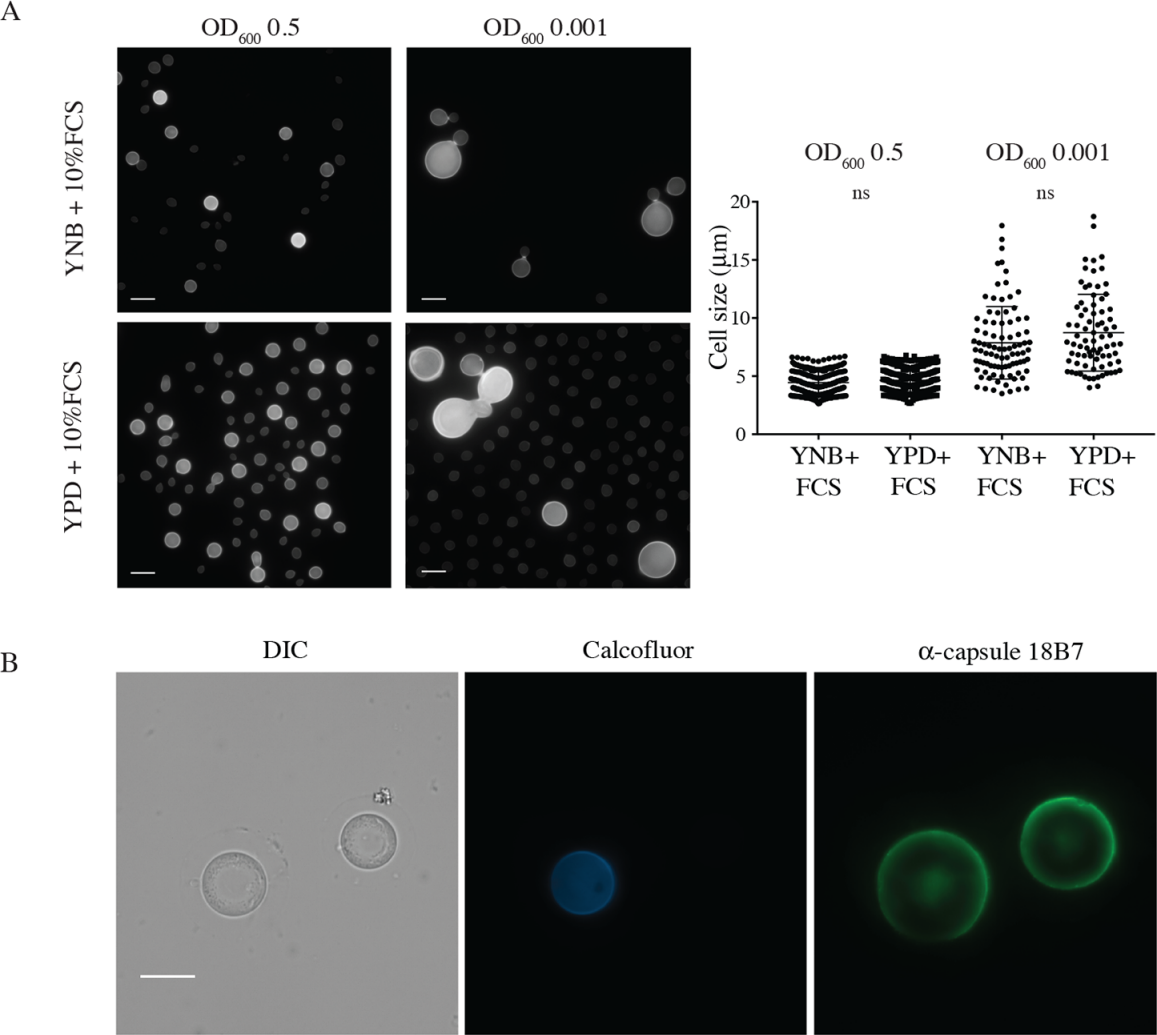
Titan cells are primed by YNB and can be maintained in 10%FCS. A) H99 cells pre-cultured in either YPD or YNB were washed 6 times in 1xPBS and then inoculated at the indicated OD in 10% FCS (3 days, 37°C, 5%CO_2_). Representative micrographs and cell size quantification are shown. B) Titan cells were induced for 24 hr, enriched for cells >11μm, and stained with CFW (10 μg/ml), then returned to inducing conditions for 48 hr. Representative micrographs show CFW (chitin) and mAb 18B7-labeled capsule. Scale bar = 10 μm.

Our *in vitro* induction protocol is a two-step process: cells are first incubated under minimal media conditions, and then induced to undergo the yeast-to-Titan switch via exposure to FCS. FCS is commonly used to induce capsule following growth in rich medium [28], suggesting that the pre-growth condition is relevant for Titanisation. YNB-grown cultures reach lower OD than YPD-grown cultures after 16 hours (mean OD_600_ = 2.733 ± 0.5608 YNB vs 16.67 ± 4.91 YPD). Given the observed impact of subsequent inoculation density on Titanisation (Fig 1C), we tested whether changes in secreted factors dependent on overnight culture cell density might repress Titanisation. YPD-grown cells were washed 6 times in PBS to remove residual exogenous compounds and incubated in 10% HI-FCS at OD_600_ = 0.5 or 0.001 (Fig 4A). Titan cells were not observed in either YPD or YNB-pre-grown cultures at OD_600_ = 0.5. At OD_600_ = 0.001, washed YPD-grown cells produced large cells at rates similar to YNB-grown cells (p>0.99). However, where YNB-grown Titan cells produce disproportionately small daughters, similar in size to yeast daughters, YPD-grown large cells frequently produced large buds, proportional to the large mother cell and not consistent with previous descriptions of *in vivo* Titan cell behavior [4, 29]. YPD-grown mother-daughter pairs also tended to be dysmorphic, with defects in cytokinesis, atypical of the reported morphology of *in vivo* Titan cells (Fig 4A)[4, 20].

Having established that Titan cells can be generated from haploid cells *in vitro*, we next tested whether Titan cells can produce Titan progeny. H99 haploid cells were pre-grown in YNB and then induced to form Titan cells overnight. After 24 hours, cells were passed through an 11 μm filter and the cells >11 μm were collected, stained with calcofluor white (CFW), and returned to fresh inducing conditions at OD_600_ = 0.001. After 72 hours, the heterogeneous population included Titan cells with robust capsule stained with both high levels of CFW and no CFW (Fig 4B).

These data suggest that nutritional pre-culture and induction cell density influence the generation of Titan cells, and that Titan cells can be stably maintained *in vitro*.

### Titanisation can be induced by bacterial cell wall components

**Fig 5:**
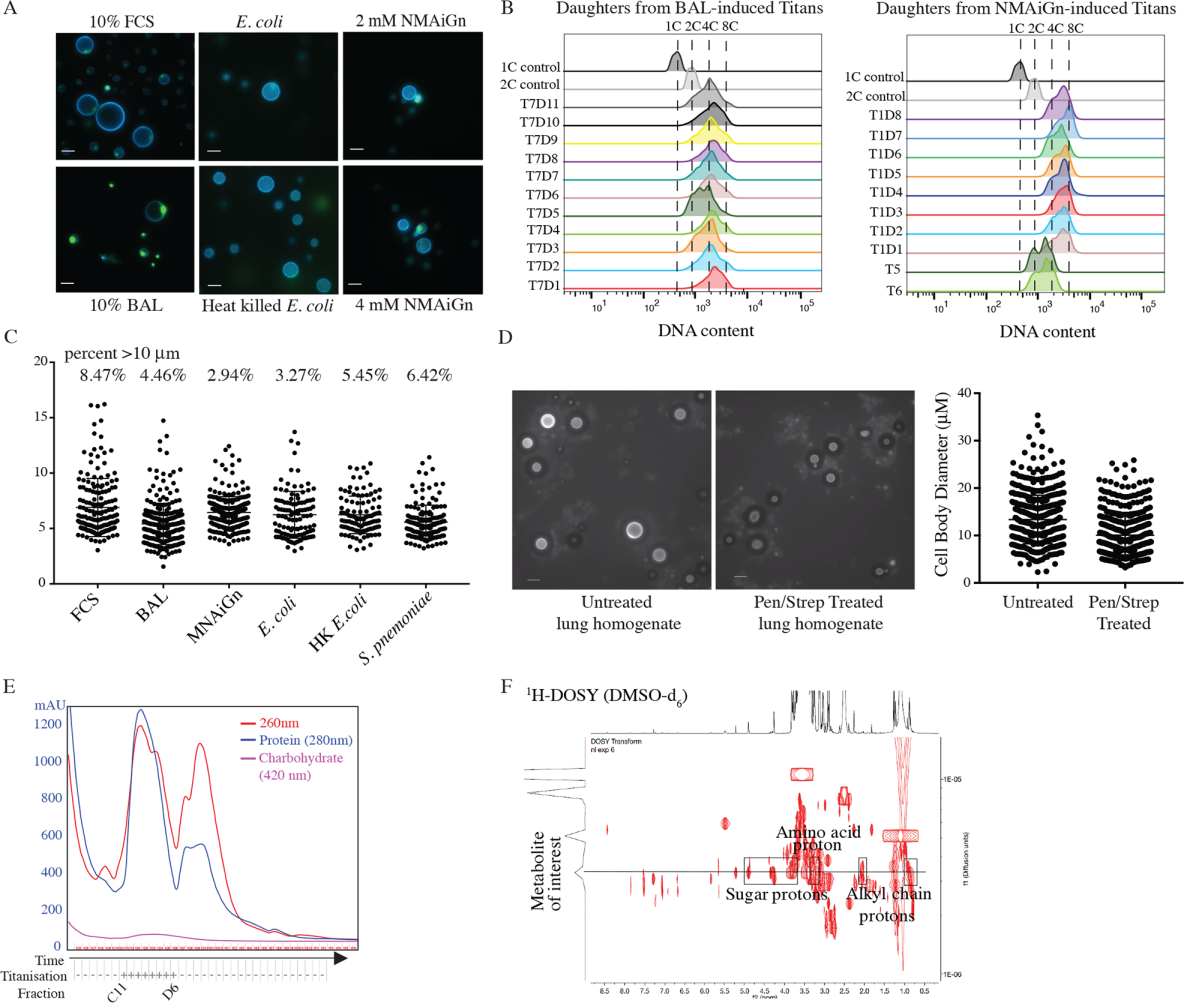
Titan cell induction is mediated by host relevant ligands and pre-culture condition. A) H99 cells pre-cultured in YNB were induced for Titans following 3 days growth in 10% FCS, 10% BAL, or 10 μM or 20 μM N-Acetylmuramyl-L-alanyl-D-isoglutamine (NMAiGn) as indicated. Representative micrographs are shown for live cells (CFW, SytoxGreen). Scale bar = 10 μm. B) Daughters arising from Titan mothers induced using BAL (left, T7) or NMAiGn (right, T1, T5, T6) were isolated and allowed to proliferate for 72 hr at 30°C on YPD agar prior to fixation and staining for DNA content (DAPI). Populations were analysed by flow cytometry. C) Quantification of cells induced by various conditions (FCS, BAL, MNAiGn, *E*. *coli*, *S*. *pneumonia*) (n>300). D) Lung homogenates from mice unexposed or exposed to Pen/Strep for 7 days prior to *C*. *neoformans* inhalation infection. Representative micrographs (CFW, scale bar = 20 μm) and quantification are shown. n>500 for each condition. E,F) FCS was fractionated by size exclusion chromatography (E) and the resulting fractions were tested for capacity to induce Titan cells. F) Analysis by ^1^H NMR and ^1^H-^13^C HSQC revealed peaks consistent with sugar and amino acid structures.

Titan cells have been identified in the host lung and brain, but have not been observed circulating in the blood or CNS. To test the impact of host-relevant inducing compounds, we asked whether murine Bronchial Alveolar Lavage (BAL) extract could induce Titan cells. When 10% BAL was used in place of FCS, we observed large polyploid cells similar to FCS-induced Titans (Fig 5A). Daughter cells arising from BAL-induced Titans were micro-dissected and cultured as described above for FCS-induced daughters. BAL-induced Titan daughters also exhibited a shift in base ploidy to 2C and 4C, with daughters arising from the same mother showing a range in base ploidy (Fig 5B). Quantification of FCS and BAL-induced Titan cells showed statistically similar populations (Fig 5C). Therefore, BAL fluid and FCS share the same capacity to induce the yeast-to-Titan transition.

BAL extract contains lung-resident bacteria, a normal component of the host microbiome and bacterial cell wall has been identified in FCS as a ligand for *C*. *albicans* morphogenesis [30–32]. To model the role of the host microbiome on Titanisation, we tested the impact of co-culture with gram-negative *Escherichia coli* and gram-positive *Streptococcus pneumoniae* for Titan induction. Both share a peptidoglycan cell wall, while gram-negative bacteria additionally have a lipopolysaccharide coat. Co-culture of YNB-grown *C*. *neoformans* and either live or heat-killed *E*. *coli* or live *S*. *pneumoniae* was sufficient to induce Titan cells after 24 hr (Fig 5A and 5C).

We tested the *in vivo* relevance of the host microbiome on Titan cell induction by comparing fungal cell size in the lungs of infected mice to fungal cell size in the lungs of mice pre-treated with antibiotic water for 7 days. There was no difference in fungal CFUs between treated and untreated mice (p>0.085). Whereas bacteria could be cultured on LB at a low level from the homogenized lungs of untreated mice, bacteria in the lungs of treated mice was below the threshold of detection (S3A Fig). We cannot rule out the presence of non-culturable bacteria in the lungs of these mice. We examined lung homogenates (Fig 5D) and histology (S3A Fig) for evidence of Titanisation. Although large cells were observed in homogenates from both treated and untreated mice, there was a significant reduction in median cell size for treated mice (Fig 5D, (untreated = 12.65 ± 5.11 vs. treated = 9.32 ± 4.14; n>500 p<0.0001)) and a 32.9% reduction in cells >10 μm, suggesting that antibiotic treatment reduced the degree of Titanisation in the lungs, possibly through perturbation of the host environment. Exposure of *C*. *neoformans* to antibiotic had no impact on Titanisation *in vitro* (S3B Fig).

Together, these data suggest that host-relevant factors in both FCS and BAL modulate *C*. *neoformans* Titanisation and suggest that bacterial factors influence *C*. *neoformans* morphogenesis. We therefore aimed to determine the minimum components of FCS necessary to trigger large cells. HI-FCS was fractionated by size exclusion chromatography, and YNB-grown H99 cells were incubated in 10% compositions of each fraction in 1xPBS. Large cells (>10 μm) were observed in cultures incubated with fractions from wells C11-D11, matching a large peak that eluted after 11 min (Fig 5E, S3C Fig). Comparison to size standards suggested that compounds in this peak are in the range of 500 Daltons. We further fractionated the pooled sample by HPLC and tested the fractions for inducing activity. Analysis by ^1^H-NMR and DOSY suggested a complex mixture of at least 9 different compounds (Fig 5F). NMR data suggested the presence of a metabolite with a sugar component (δ_H_ 4.89, 4.32/4.30, 4.27, 3.79 and 3.70 ppm) and an alkyl chain (δ_H_ 2.08, 2.03, 0.87ppm). Additionally, ^1^H NMR and ^1^H-^13^C HSQC experiments exhibited a methylene (δ_H_ 3.12 ppm, 52.4 ppm) likely to be located in alpha conformation to a carbonyl and an amino group, which suggested the presence of an amino acid substructure in this metabolite. These features are consistent with peptidoglycan structures. Coupled with our observation that bacterial cell wall from both gram-positive and gram-negative cells is capable of triggering Titanisation, we hypothesized that this metabolite might represent a bacterial peptidoglycan.

Muramyl tetrapeptides (MTP) are peptidoglycan subunits common to the cell walls of Gram negative, Gram positive, and myco-bacteria. Muramyl tetrapeptides consist of an ether of N-acetylglucosamine (GlcNAc) and lactic acid (MurNAc), plus a species-specific tetrapeptide. MTPs act as signaling molecules in both mammalian and fungal cells by binding Leucine Rich Repeat (LRR) domains in target proteins, including mammalian NOD receptors, expressed on phagocytes and epithelial cells in the lung, and *C*. *albicans* adenylyl cyclase [32, 33]. MTP and its derivatives were identified as potent inducers of the yeast-to-hyphal transition in *C*. *albicans* following spectroscopic analysis of serum, which was shown to contain low levels of bacterial cell wall component [32]. The synthetic Muramyl Dipeptide (MDP), *N*-Acetylmuramyl-L-alanyl-D-isoglutamine (NMAiGn), is structurally similar but not identical to MTP. ^1^H NMR analysis of NMAiGn was consistent with the peptidoglycan peaks identified in the FCS fractions. Therefore, we tested the capacity of MNAiGn to influence *C*. *neoformans* morphogenesis. Titan cells were induced using 2 mM or 4 mM NMAiGn (the concentration sufficient to trigger the yeast-to-hyphal switch in *C*. *albicans*). Cells incubated with NMAiGn exhibited limited proliferation; however, cells >10 μm were present at both concentrations, consistent with a yeast-to-Titan switch (Fig 5A and 5C).

Individual large cells were isolated by microdissection and allowed to proliferate for 17 hr at 30°C on YPD agar. Of 6 large cells isolated, all 6 proliferated to form colonies. For four of these colonies, individual daughters, distinguishable through their reduced size relative to the mother, were again isolated and allowed to proliferate for a further 72 hrs. The remaining 2 colonies (T5, T6) from the original large cells were analysed in aggregate. Each of the resulting lineages was analysed by flow cytometry for ploidy. In NMAiGn-induced daughter cells, we observed an overall increase in ploidy, with the majority of colonies arising from individual daughters having a 4C base ploidy. A representative lineage is shown in the right panel of Fig 5B. Aggregate samples (T5, T6) were more heterogeneous and included 2C and 4C cells, consistent with diploid daughter lineages (Fig 5B right). Together, these data demonstrate a role for peptidoglycan such as MDP during *in vitro* Titanisation and suggest that bacterial components influence Titanisation *in vivo*.

### The cAMP pathway positively regulates *in vitro* Titanisation

**Fig 6:**
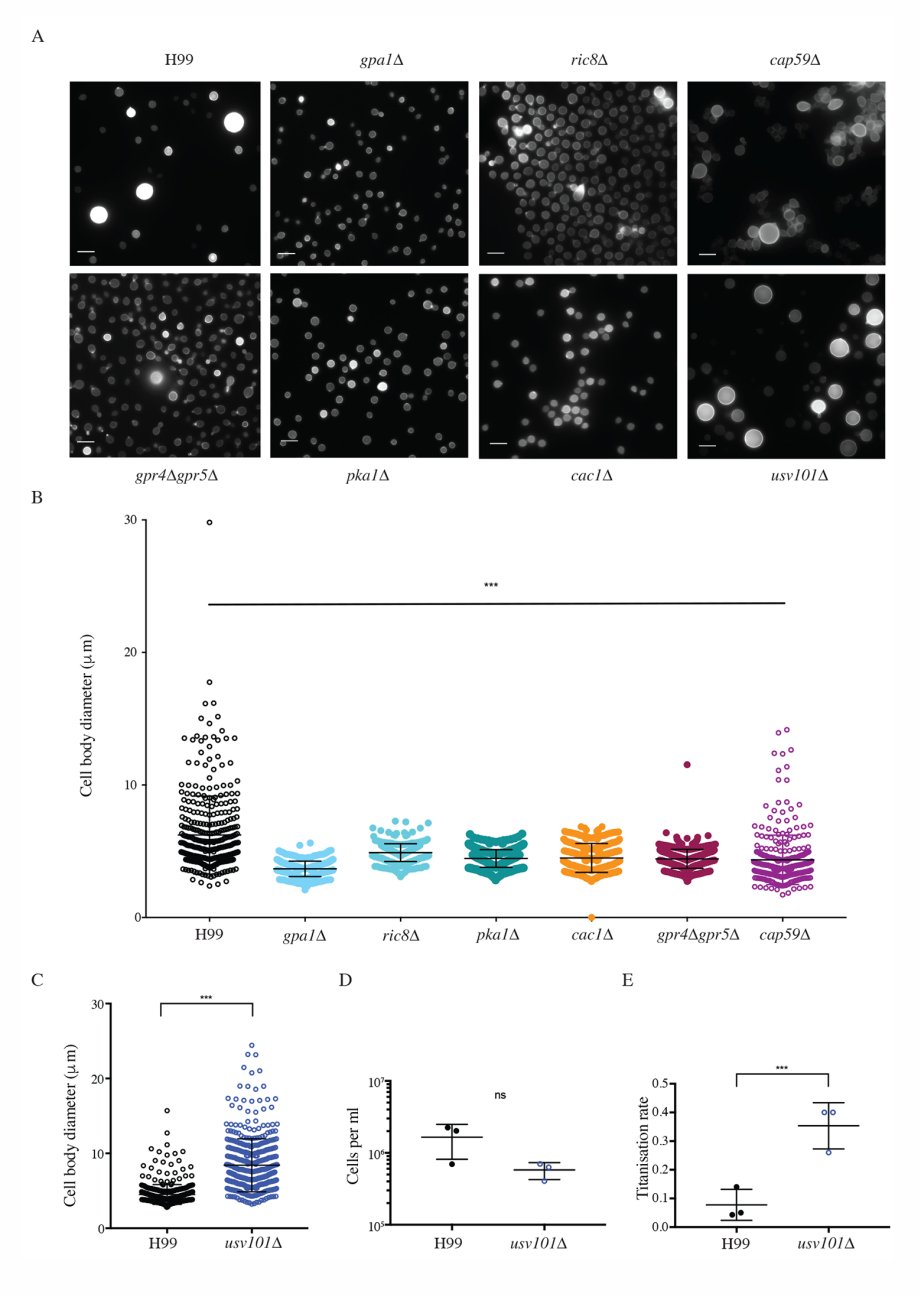
The cAMP/PKA pathway and the Usv101 transcription factor influence *in vitro* titanisation. A,B) Wild-type, *gpa1Δ*, *ric8Δ*, *gpr4Δgpr5Δ*, *pka1Δ*, *cac1Δ*, and *cap59Δ* cells were incubated under Titan inducing conditions. A) Representative micrographs for fixed cells are shown (CFW). Scale bar = 10 μm. B) Cell size was quantified for the indicated strains. Significance was assessed using Kruskal-Wallis and Dunn's multiple comparisons (relatively to H99, p<0.0001 for all strains, n>300). C) Wild-type and *usv101Δ* cells were incubated under the indicated conditions. Representative micrographs for fixed cells are shown (CFW). D) Cell size was quantified for wild type and *usv101Δ* cells induced in YNB+10% FCS (p<0.0001). E,F) H99 and *usv101Δ* Titan cells were induced for 24 hrs, enriched for cells >11μm, normalized to 10^3^ cells per ml, then returned to inducing conditions for 48 hr. Total cell populations and cells >11μm were then counted by haemocytometer as a measure of E) cell proliferation (p = 0.116) and F) Titanisation rate (p = 0.008) (triplicate biological replicates). Significance was assessed using Mann Whitney U in all cases.

Muramyl dipeptide is thought to interact directly with the LLR domain of adenylyl cyclase, and the cAMP signal transduction cascade is believed to regulate Titanisation *in vivo* [20, 22, 32]. However, addition of exogenous cAMP at levels sufficient to induce capsule failed to induce Titan cells in either YPD or YNB-grown cultures. The avirulence of mutants deficient in cAMP signal transduction has precluded direct testing of this model [20, 34, 35]. We therefore examined the influence of *GPA1*, *CAC1*, and *PKA1*, as well as *RIC8*, a Gα Guanine Nucleotide Exchange Factor (GEF) for Gpa1, on *in vitro* Titanisation [36]. Strains deficient in each of these genes failed to generate large cells in our assay (Fig 6A and 6B). The G-protein coupled receptor Gpr5 is required for Titanisation, and the *gpr4Δgpr5Δ* strain exhibits a significant reduction in Titan cell production *in vivo* [7, 20]. We likewise observed a decrease in the frequency of Titan cells *in vitro* in the *gpr4Δgpr5Δ* strain (Fig 6A and 6B). Consistent with the incomplete defect observed *in vivo* [20], rare Titan cells could be observed *in vitro* for this strain (Fig 6A and 6B). Although *cap59Δ* cells were smaller overall, we observed no specific defect in the capacity of the capsule deficient strain to form Titan cells, ruling out that Titan defects in this pathway are related to defects in capsule synthesis (Fig 6A and 6B). Together, these data demonstrate that *in vitro-*induced Titan cells are regulated via a similar pathway to *in vivo* Titan cells.

### Usv101 negatively regulate *in vitro* Titanisation

The C_2_H_2_ transcription factor Usv101 is a master regulator of *C*. *neoformans* pathogenesis that negatively regulates capsule and acts downstream of Swi6, a regulator of cell cycle progression [37–39]. Usv101 is additionally predicted to regulate Gpa1 but is not itself directly influenced by cAMP[37]. We therefore investigated the role of Usv101 in *in vitro* Titanisation. Consistent with its role as a negative regulator, *usv101Δ* produced significantly more and larger titan cells *in vitro* compared to the H99 parent (Fig 6A and 6C; 39.25±6.45%, p<0.0001). No difference in cell size was observed during YNB pre-culture (H99: 5.918±0.8126; *usv101Δ*: 5.674±0.9084; p = 0.017). Titans evade phagocytosis and are predicted to drive dissemination through the production of daughter cells, but inactivation of *USV101* increases phagocytosis of yeast-phase cells [37]. We hypothesized that Titan *usv101Δ* cells might fail to produce daughter cells required to drive dissemination to the brain. We therefore measured the relative production of daughter cells by purified cultures of Titan cells from H99 vs. *usv101Δ* inocula. When Titanized cells were taken as the starting culture, no difference in the total number of cells produced over time was observed for the two strains (Fig 6D, proliferation rate, p = 0.116). However, there was a significant difference in the Titanisation rate (proportion of yeast vs. Titan) between the two strains, with *usv101Δ* Titan daughters 4.5 times more likely than H99 Titan daughters to form new Titan cells (Fig 6E, H99 Y/T- = 0.0777 ± 0.0312; *usv1010Δ* Y/T = 0.353 ± 0.0467; p = 0.008). These data suggest that the increased capacity of the *usv101Δ* mutant to form Titan cells over time may contribute to the previously reported reduced dissemination and reduced virulence of this strain *in vivo* [37].

### *In vitro* Titanisation can predict *in vivo* Titanisation

**Fig 7:**
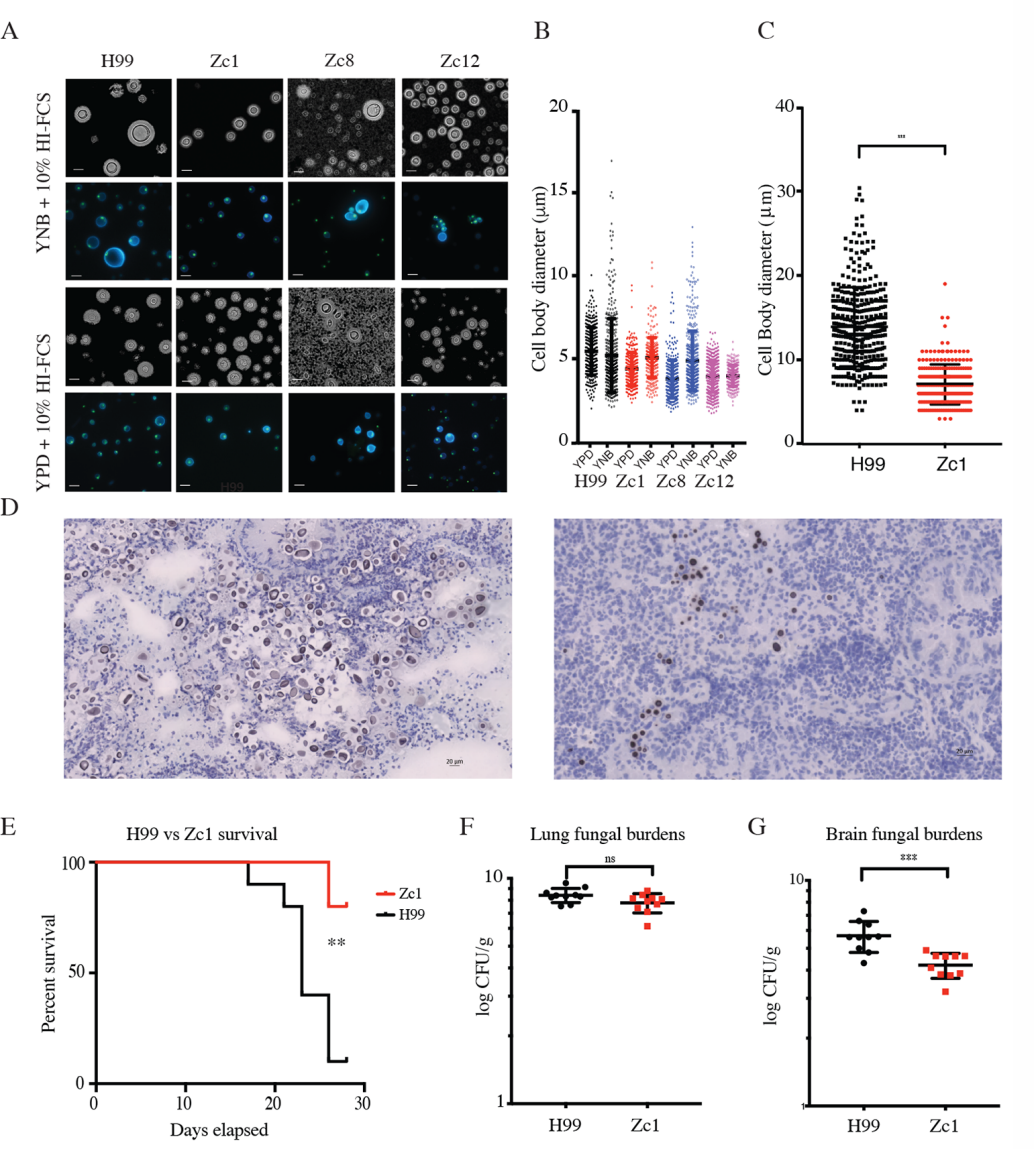
*In vitro* Titanisation predicts *in vivo* outcome. A,B) H99, Zc1, Zc8, and Zc12 clinical isolates were pre-cultured in either YPD or YNB and then inoculated OD_600_ = 0.001 in 10% FCS (3 days, 37°C, 5%CO_2_). A) Representative micrographs of live cells stained with CFW and SytoxGreen or counterstained with India ink and cell size quantification are shown. B) Representative lung histology from mice infected under the indicated conditions and sacrificed on day 7. Cell size was measured for each condition (n>500 cells for each condition). C,D) Quantification of cell size (n>300; p = 0.001) and histology from mice infected with H99 or Zc1 for 7 days prior to *C*. *neoformans* inhalation infection. Scale bar = 20 μm. E,F,G) Balb/C mice (n = 10 per group) were infected with either Zc1 or H99 intra-nasally and followed for 28 days. E) Kaplan-Meyer survival curve (humane endpoint) (p = 0.007). CFUs for lung (F, p = 0.907) and brain (G, p<0.0001) were measured on day of cull.

We examined the capacity of non-H99 strains to produce Titan cells *in vitro*. Titanisation has not been reported for *Cryptococcus gattii*, and no increase in cell size was observed for *C*. *gattii* isolate R265 (S4A Fig). Next, we screened 62 environmental and clinical *C*. *neoformans* isolates representing VNI, VNII, and VNB clades [40]. Strains were classified as Titanising, non-Titanising, or Indeterminate (S4B Fig). A wide variety of cell sizes were observed in response to inducing conditions, and representative isolates Zc1, Zc8, and Zc12 (VNI clade) are shown in Fig 7A. After growth on YPD, these isolates are morphologically similar, and are capsular, thermotolerant, and melanising, comparable to H99, but exhibit distinct Titanisation profiles (Fig 7A, S4C Fig). Across the 62 isolates, we observed a wide range in Titanisation capacity in both clinical and environmental isolates from each clade, including clinical strains with defects in Titan cell production (Zc1, Zc12; Fig 7A), and environmental strains that produced Titan cells (S8963, Ze14 (VNB-B); S4B Fig). We also observed non-H99 clinical strains that Titanised (Zc8; Fig 7A) and environmental strains that did not (Ze18 (VNB-B), S4B Fig). Together, these data suggest that the yeast-to-Titan switch is a conserved morphogenic transition that can occur across the *C*. *neoformans* var. *grubii* species complex, but that individual isolates within each clade exhibit different capacities to form Titans.

Finally, we validated the capacity of our *in vitro* assay to predict *in vivo* outcome in a murine inhalation model of infection, the current gold standard for Titanisation analysis, using the type strain H99 and a clinical isolate predicted not to form Titans, Zc1 (Fig 7A and 7B, p = 0.0184). Mice infected with Zc1 or H99 were observed for 7 days and then sacrificed, and the lungs and brain were collected. Notably, there were clear differences in lung pathology at 7 days despite comparable lung CFUs (S4D Fig). Lungs from H99-infected mice exhibited large lesions or granuloma in contrast to lungs from Zc1-infected mice, which exhibited fewer or no apparent lesions or granuloma (S4E Fig). Histology also revealed foci of encapsulated fungi in the lungs of H99-infected mice, with heterogeneous cell size including both Titan (>15 μm) and yeast (<10 μm) cells (Fig 7C and 7D). In contrast, histology of the lungs of Zc1 infected mice revealed disseminated infection, with encapsulated yeast distributed throughout the lung parenchyma. The population was more uniform in size, with the vast majority of cells less than 10 μm (Fig 7C) (mean 13.9±4.6 vs. 7.08±2.38; p<0.0001).

Given the differences in histo-pathology, we measured relative pathogenicity using a long-term survival assay in Balb/C mice. H99 was significantly more virulent than Zc1 (p = 0.007, Fig 7E). No significant difference was observed in lung CFU on day of sacrifice (p = 0.0575, Fig 7F), however H99-infected mice exhibited significantly higher CFUs in the brain (p = 0.0005, Fig 7G). A similar trend was observed when the same analysis was performed in C57Bl/6J mice (S4G Fig). This is consistent with previous observations of differential tropism when Titan cells are present [19].

**Fig 8:**
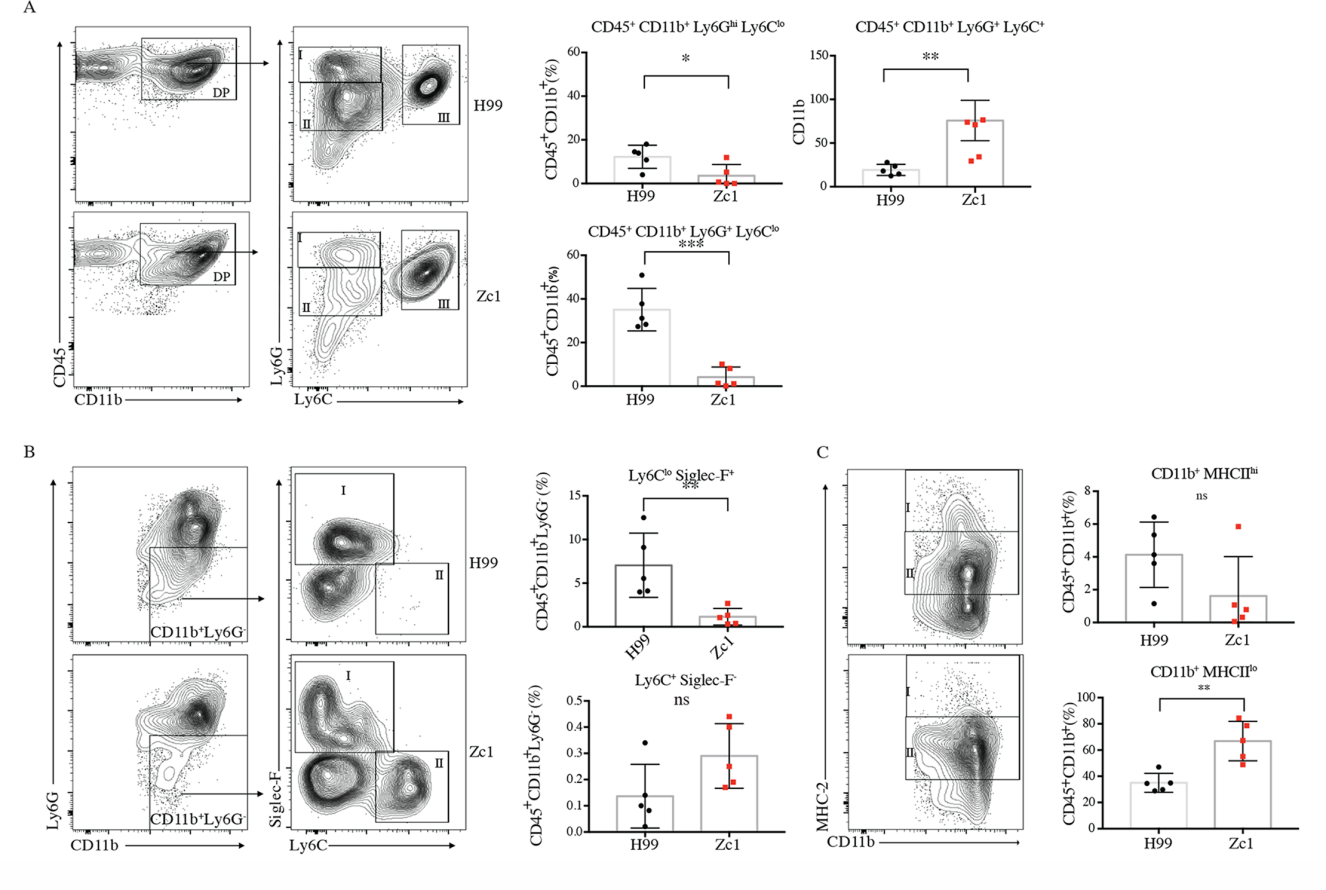
Zc1 and H99 infections differentially impact host immune response. FACS analyses for immune cell recruitment to the lungs for mice infected with H99 or Zc1 (day 7 p.i., n = 5 per group). A) Concatenated analysis for 5 mice under each condition were gated for CD45^+^ CD11b^+^ populations and then analysed for Ly6G and Ly6C markers. Indicated populations of CD45^+^ CD11b^+^ cells in individual mice were analysed for the two conditions (* p = 0.0299; **p = 0.0079; ***p<0.0002). B) Indicated populations of CD45^+^ CD11b^+^ Ly6G- cells in individual mice were further analysed for SigLecF and Ly6C markers (** p = 0.0085). C) Indicated populations of CD45^+^ CD11b^+^ cells in individual mice were further analysed for MHCII markers (** p = 0.0027).

Titanisation is associated with altered immune response and increased dissemination to the brain [7, 19]. We therefore investigated the impact of the two strains on immune response in the lungs on day 7 post-infection. With the distinct nature of capsules between Titanising and non-Titanising isolates, we speculated that these two groups might express unique PAMPs and thus differ in their interactions with immune cells. For this purpose, we focused our attention on cells of myeloid origins. The total number of CD45 cells was not significantly different (p = 0.158). We observed recruitment of leukocytes into the lungs of both groups of mice, primarily comprised of CD11b^+^ granulocytes (Fig 8A and 8B). Three distinct subsets characterized by Ly6G and Ly6C expression were observed: mature neutrophils (Ly6G^hi^, gate I) and two populations of immature Ly6G^int^ neutrophils expressing Ly6G^lo^ (gate II) and ly6C^hi^ (gate III) (Fig 8A). While H99-infected mice had more mature neutrophils (12% vs. 3.7%; p = 0.0299, gate I) as well as Ly6C^lo^ immature neutrophils (37.6% vs 5.2%; p = 0.0002, gate II), Zc1-infected mice exhibited significantly higher percentage of the Ly6Chi immature neutrophil pool (Fig 8A, 76.1% vs 19.5%; p = 0.0079, gate III). The remaining CD11b+ non-neutrophil compartment contained, amongst others, eosinophils (SiglecF^+^) and monocytes (Ly6G^-^ Ly6C^hi^) (Fig 8B). Eosinophils were found to be higher in H99-infected mice (53.5% vs 28.8%, p = 0.0085; Fig 8B, gate I) and monocytes were elevated in Zc1-infected mice, although the difference did not reach significance (Fig 8B, gate II; p = 0.3095). We also observed differences in the frequency of cells expressing the MHC-II molecule involved in antigen presentation (Fig 8C). Although CD11b^+^ MHC-II^hi^ cells (gate I) were present in both groups, mice infected with H99 displayed an increasing trend of this subset, while Zc1-infected mice had a significant increase in CD11b^+^ MHCII^lo^ cells (p = 0.0079, gate II) (Fig 8C).

Taken together, these data suggest that our *in vitro* assay accurately predicts *in vivo* Titanisation and that isolates that form Titan cells drive quantitatively distinct immune responses from those that do not.

## Discussion

The yeast-to-Titan transition is a host-specific morphogenic switch that can influence disease outcome. Titan daughters have altered stress resistance compared to their mother cells, and the presence of Titans is associated with altered immune status [6–8]. Despite their importance, mechanisms underlying Titanisation have been challenging to dissect due to the lack of a reproducible *in vitro* induction protocol. Here we present a rapid, robust *in vitro* induction protocol that generates cells with all the properties of *in vivo* Titan cells, recapitulates previously identified regulators (Gpr4/Gpr5), directly confirms the role of cAMP pathway elements (Gpa1, Cac1, Pka1) and identifies new regulators (Ric8, Usv101). The assay further accurately predicted an *in vivo* defect in Titanisation by a clinical isolate, Zc1.

We define *in vitro* Titans as those >10 μm, and show that in our assay approximately 15% of H99 cells form Titans within three days (Fig 1). Additionally, we show that purified Titan cultures produce heterogeneous cell populations, including new Titan cells (Figs 3B and 4B). Therefore, our growth conditions are sufficient for the induction and maintenance of Titan cell cultures. Whereas budding haploid yeast undergo symmetric DNA division and produce uniform populations of haploid daughters that are proportional in size, *in vivo-*derived polyploid Titan cells produce small (5μm) haploid, aneuploid, or diploid daughters [3, 6]. Likewise, we show that *in vitro* Titans are uninucleate and divide DNA asymmetrically (Fig 3). We also distinguish between *bona fide in vitro* Titan cells and Titan-like cells induced from rich media pre-culture, which tended to form large buds and accumulated chains of large cells that failed to complete cytokinesis (Fig 4A).

Some definitions of Titan cells include capsule when determining Titan cell size (>30 μm) [23]. During yeast phase growth, there is a demonstrated a relationship between capsule size and the length of the G1 phase of the cell cycle [21, 41]. In these reports, capsule size increases with cell body size and defects in cell cycle control influence capsule expression. Consistent with this, we observed that ploidy influences capsule (Fig 1E): Titan cells induced from diploid parents were significantly larger than those from haploid parents when capsule was taken into account, but the difference was not significant when cell body alone was examined. We also show that under Titan inducing conditions the capsule:cell body ratio changes as cells cross the 10 μm threshold (S1C Fig). For example, for the cells shown in Fig 1A, the yeast cell is 4.98 μm with an 18.49 μm capsule (ratio of 3.70), while the Titan cell is 21.9 μm with a 37.17 μm capsule (ratio of 1.69). This is consistent with observations that Titan capsule synthesis is distinct from yeast phase capsule synthesis and suggests a difference in the regulatory control of the two phenotypes [18, 23]. Future work will examine the specific impact of *in vitro* Titan inducing conditions on capsule regulation and structure.

*C*. *neoformans* is a globally distributed environmental fungus, and the mammalian lung is not thought to be a reservoir for *C*. *neoformans*[42]. Rather, our data suggest that the host lung may serve as a niche for bacterial-fungal interactions that mediate pathogenesis. Titan cells are observed in the host lung, an environment with a poorly understood but complex microbiome [31], and BAL can replace FCS as the inducing compound. BAL samples from healthy individuals are positive for bacterial 16S RNA (8.25 log copies/ml) and FCS contains 0.1–0.5 μM Muramic acid, a marker of bacterial peptidoglycan[30, 32]. We identified structures consistent with peptidoglycan in serum fractions capable of inducing Titanisation. Antibiotic treatment that reduced culturable bacterial lung burdens reduced Titan induction in a murine inhalation model, and co-culture of live or heat-killed *E*. *coli* or live *Streptomyces pneumoniae* (a dominant genus of the healthy lung) with *C*. *neoformans* led to Titanisation (Fig 5A, 5C and 5D)[30]. Exposure of primed cells to NMAiGn, a synthetic version of the bacterial cell wall component MDP, was sufficient to induce Titan cells (Fig 4A and 4B). MDP and its derivatives are known to activate cAMP-mediated morphological transitions in *Candida albicans* and other ascomycetes[32]. Together, these data point to a conserved mechanism for bacterial-fungal interactions underlying morphological transitions and highlight the importance of polymicrobial interactions for understanding *Cryptococcus* pathogenesis, adding to increasing importance of the host lung microbiome in health and disease [31, 43].

There are some differences between our findings and the published literature. First, *in vivo* generated Titan cells achieve extreme cell size within three days (Fig 7C)[3, 19]. Single cell analysis of our *in vitro* Titan cells did not identify cells exceeding 30 μm after 3 days, however we did observed cells > 60 μm after 7 days continuous culture (S1A Fig). In addition, we identify a subpopulation of cells with very thin cell walls and altered cell shape, which we term Titanides. These cells appear to be distinct from previously described *in vivo* micro-cells (<1μm, thick cell walls) [25] and typical yeast daughter cells and accumulate during *in vitro* Titan cell induction (Figs 2A, 2B and 3B). Based on their altered cell wall, Titanides are likely to differ in the exposure of host relevant ligands relative to yeast cells, and their small size may facilitate dissemination, either through phagocytosis or through increased penetration of the lower airways, similar to the dissemination of basidiospores [44]. Despite these differences in our single cell analysis, bulk analysis of total cell cultures (>10,000 cells) revealed a heterogeneous cell size population that also exhibits a wide range in cell ploidy consistent with previously reported *in vivo* Titan populations (Fig 1B). Future work will further characterize these cells and investigate the role of the various sub-populations in pathogenesis.

Second, while we observed asymmetric division of nuclear content in dividing Titan cells (Fig 3A), microscopic analysis using a mCherry-Cse1 reporter strain for DNA content of individual cells in the heterogeneous population suggested than the majority of cells are diploid or aneuploid. Additionally, FACS analysis of colonies derived from single daughter cells isolated from *in vitro* Titan mothers were aneuploid, diploid or, in some cases tetraploid, and cell size in these populations was consistent with this increased ploidy (Fig 3C, 3D and 3E; S2 Fig). In contrast, colonies arising from a limited number of *in vivo* Titan mothers were comprised of primarily haploid or aneuploid cells [6]. However, Gerstein et al. also report that 25% of independent *in vivo-*derived Titan daughters were diploid. This highlights an important aspect of Titan cell biology that has proved challenging to study. Titan cells are thought to allow phenotypic diversity through the generation of aneuploid daughters, with increased access to the fitness landscape as a result of changing gene dosage[6]. Current models suggest that uninucleate, highly polyploid mothers bud off haploid or aneuploid daughters, requiring asymmetric DNA division via an unknown mechanism. Efforts to understand the molecular mechanisms underlying this unusual process will benefit from an *in vitro* model, and our data already suggest that the diversity of these daughter cells is greater than previously described.

Our *in vitro* model offers some initial insights into the underlying molecular mechanisms regulating Titanisation *in vivo*. First, *in vitro* Titans form following exposure to low nutrient conditions and dependent on cell density, suggesting that Titanisation occurs in response to a two-part Prime-Induce signal. *C*. *neoformans* growth in minimal media is known to alter the expression of secreted proteins relative to YPD and influences stress resistance through transcriptional and post-translational changes [45, 46]. Exposure to serum is a known signal for capsule induction via the cAMP/PKA pathway [28]. Interestingly, Zaragoza et al. have previously reported that co-incubation of *C*. *neoformans* in serum + Sabouraud-Dextrose can increase the average cell size (up to 9 μm), yet represses the influence of serum on capsule [28]. Additionally, serum is a potent inducer of the morphogenic switch from yeast to hyphae in *C*. *albicans* and *Yarrowia lipolytica* via cAMP and Ras1 [32, 47, 48]. In the case of *C*. *albicans*, bacterial MDP was identified as the essential component of serum driving the activation of Cyr1 and cAMP signaling. In *C*. *neoformans*, previous work has strongly suggested a role for the cAMP signal transduction cascade in Titanisation *in vivo* [20]. Our finding that bacterial MDP similarly induces the Yeast-to-Titan transition suggests a similar signaling cascade may be in place.

Because *gpa1Δ*, *pka1Δ*, and *cac1Δ* strains are rapidly cleared from the host lung, direct testing of their role in Titanisation was not previously possible *in vivo* [20, 34, 35]. Here, we confirm this model through direct demonstration that cells deficient in adenylyl cyclase activity, but not capsule biosynthesis, fail to form Titans *in vitro* (Fig 6A and 6B). Despite the requirement for adenylyl cyclase activity, constitutive activation is not sufficient to induce Titans. The addition of exogenous dcAMP does not induce Titanisation nor does it restore Titanisation to cAMP/Pka pathway mutants in our *in vitro* assay. Additionally, hyper-activation of the pathway using GAL-inducible *PKA1* and *PKR1* constructs produces a heterogeneous population of both large, polyploid cells and yeast phase cells after 48hr [22]. Similarly, expression of a constitutively active version of the Gα protein Gpa1 doubles the percent Titan cells *in vivo* [20]. In each of these cases, Titan cells make up a fraction of the total cell population. These data suggest that Titan induction via cAMP/Pka1 interacts with metabolic, transcriptional or post-translational priming of individual cells to determine cell fate upon exposure to inducing conditions such as serum, resulting in a heterogeneous population.

In addition to positive cAMP regulation, we identify the transcription factor Usv101 as a negative regulator of Titanisation (Fig 6). Although *USV101* has been shown to be dispensable for virulence, murine infection results in delayed dissemination to the brain and is characterized by pneumonia rather than meningitis [37]. Gish et al. demonstrated that *usv101Δ* fails to cross an *in vitro* blood-brain barrier model and *usv101Δ* yeast are phagocytosed more readily than wild type cells, partially explaining the altered virulence of this strain. This is somewhat surprising, as together with capsule-independent direct crossing, phagocytosis by trafficking macrophages is thought to facilitate transmigration of the BBB [49]. Here, we show that cells lacking *USV101* form Titan cells at a high frequency compared to the parental strain (Fig 6A and 6C) and that these *usv101Δ* Titans themselves form Titans at a higher rate than wild-type cells (Fig 6E). We suggest that increased Titanisation and decreased availability of non-Titan fungal cells inhibits dissemination of this strain outside of the lung.

In addition to its influence on capsule, Usv101 is predicted to act in parallel to the cAMP pathway and downstream of the cell cycle regulator Swi6. The interaction between cell cycle and pathogenicity factor expression is an emerging theme in *C*. *neoformans* biology: Recent work has also highlighted cell cycle regulation of pathogenicity factors [39] and the cyclin Cln1 has been shown to regulate capsule and melanin, both of which are regulated by cAMP and negatively regulated by Usv101 [37, 38, 41, 50].

Finally, the clinical isolate Zc1 and the clinical type strain H99 elicited distinct immune responses. While both H99 and Zc1 strains induced leukocyte recruitment into the lungs, granulocytes predominated the response, and these could be clustered into three unique subsets (a mature neutrophil subset (Ly6G^hi^) and two distinct immature neutrophil subsets (Ly6C^lo^ and Ly6C^hi^)). H99 infection was associated with increased frequency of mature neutrophils and the LyC6^lo^ immature neutrophil subset, whereas the Ly6C^hi^ immature neutrophils were dominant during infection with the Zc1 isolate. It is not known whether these cells express different effector functions and what their polarization state is, and thus more work is required to better understand if they mediate protection or susceptibility to infection with *C*. *neoformans*. Other notable differences in immune responses involved disparate frequencies of eosinophils and monocytes recruited during infection. Increased frequency of eosinophils and CD11b^+^MHC-II^hi^ was observed in mice infected with H99 relative to the Zc1 isolate while the frequency of monocytes and CD11b^+^MHC-II^lo^ cells was higher in Zc1-infected mice. Overall, Zc1-infected mice exhibited moderate lung pathology and reduced dissemination to the brain (Fig 7D–7G), suggesting that there might be qualitative differences in the immune responses driven by Zc1 vs. H99.

We note that other labs have recently reported alternate conditions for inducing Titan-like cells *in vitro*, either through incubation in SabDex+FCS+sodium azide or through exposure to hypoxia, low pH, and low nutrient conditions [51, 52] Together, these data suggest that Titanisation, like filamentation in *C*. *albicans*, is a morphogeneic transition that can be initiated in response to a variety of different stress conditions. One intriguing model for the induction of Titan cells suggests a role for the host immune system: in two studies, mouse genotype interacted with *C*. *neoformans* cell size [53] [8]. Our findings that Titanisation capacity varies across clinical and environmental isolates, as well as our data demonstrating that Titanisation can be triggered by bacterial MDP, adds additional complexity to these observations. The interaction of the host with fungal and bacterial co-infecting species is a theme of emerging importance in our understanding of fungal pathogenesis, both in the context of increased disease severity and through the inhibition of pathogenicity [43, 54, 55].

Our *in vitro* system enables *ex vivo* analysis of the role of specific host factors in Titanisation; *in vitro* dissection of the molecular mechanisms driving Titanisation; and improved understanding of the interaction between Titanisation and pathogenesis. The complex interaction of Titanisation and pathogenesis is highlighted by the findings that both Zc1, a Titan deficient isolate, and *usv101Δ*, a hyper-Titanising mutant, cause pneumonia rather than disseminated disease and meningitis [37, 40]. Zc1-infected mice exhibited significant lung pathology, including leukocyte recruitment on day 7 and high lung fungal burden on day of cull in two different models of infection (Fig 7F, S4D, S4E and S4F Fig). Our data suggest that morbidity due to pneumonia might be an important factor to consider during infection with clinical or environmental isolates. While we hypothesize that the Titanisation defect of Zc1 is primarily responsible for its delayed dissemination relative to H99, it is also possible that other differences between the two strains, or in the host response to infection, drive observed differences in pathogenesis. However, Zc1, like H99, is a VN1 clade patient isolate with no defects in the classic pathogenicity factors capsule, thermotolerance, or melaninsation. Dissemination to the brain was similar in female T_H_2-tilted Balb/C and male T_H_1-tilted C57Bl/6 mice [56, 57]. Titanisation capacity appears to be the single largest difference between Zc1 and H99, and is representative of the wide variety in Titanisation phenotypes for environmental and clinical isolates. The relative impact of Titanisation on pathogenesis and clinical outcomes is a pressing question, and future work will investigate this further, particularly in the context of immune-altered states such as neutropenia and T and B lymphocytopenia.

## Methods

### Media and strains

Strains used in this study are summarized in S1 Table. *C*. *neoformans* H99 [58], *gpa1Δ, cac1Δ[34],* and *pka1Δ*[59] were gifts from Andrew Alspaugh, Duke University, NC, USA. The *gpr4Δ gpr5Δ* [60] was kindly provided by Joseph Heitman, Duke University, NC, USA. Strains *ric8Δ* and *usv101Δ* were obtained from the Madhani 2015 collection (NIH funding, R01AI100272) from the Fungal Genetics Stock Centre and were validated by PCR and shown to phenotypically match published strains [36, 37]. *C*. *gattii* R265 [61] was provided by Neil Gow, University of Aberdeen, UK. Isolates are summarized in S1 Table. Cells were routinely cultured on YPD (1% yeast extract, 2% bacto-peptone, 2% glucose, 2% bacto-agar) plates stored at 4°C. For routine culture, cells were incubated overnight in 5 mL YPD at 30°C, 150 rpm. For Titan induction, cells were incubated overnight at 30°C, 150 rpm in 5 mL YNB without amino acids (Sigma Y1250) prepared according to the manufacturer’s instructions plus 2% glucose. Fetal Calf Serum (FCS) was obtained from either BioSera (Ringmer, UK) or Sigma, which both induced Titan cells to a similar degree. FCS was routinely stored in 5 ml aliquots at -20 to prevent repeated freeze-thaw cycles. FCS was heat-inactivated by incubation at 56°C for 30 min.

### Titan cell measurement

Cells were induced and either fixed with 4% methanol free paraformaldehyde and permeabilised using 0.05% PBS Triton-X and stained for total chitin with calcofluor white (CFW, 10 μg/ml) and DNA with SytoxGreen (Molecular Probes, 5 μg/ml) or stained live using calcofluor white (CFW, 5 μg/ml) and the cell permeable nucleic acid stain SybrGreen (Invitrogen, 0.5X). SybrGreen preferentially stains dsDNA, but has low affinity for ssDNA and ssRNA, so is not appropriate for quantitative DNA analysis. However, it does allow visualization of nucleic acid dynamics within live cells. Cells were imaged using a Zeiss M1 imager for fixed cells and either a Zeiss Axio Imager or a Nikon Eclipse TI live imager, both equipped with temperature and CO_2_ control chambers for live imaging. To visualize capsule, live cells were counterstained using India Ink (Remel; RMLR21518) or fixed and stained with mAB 18B7 and counterstained with 488-antimouse IgG. Representative images are shown. Cell diameter was measured using FIJI, with frames randomly selected, all cells in a given frame analysed, and at least three images acquired per sample for each of two independent runs, representing experimental duplicates. In all instances unless otherwise stated, n>200 cells. Statistical analyses were performed using Graphpad Prism v7. For pairwise comparisons, the Mann-Whitney test was applied. For multiple comparisons, ANOVA and Kruskal-Wallis were applied. Significance was taken as p<0.01 throughout.

### Flow cytometry for cell ploidy

Cells were fixed and stained according to the protocol of Okagaki et al 2010[3]. Briefly, cells were fixed with 4% methanol free paraformaldehyde and permeabilised using 0.05% PBS Triton-X. Cells were washed 3 times with 1x PBS and stained with 300 ng/ml DAPI. Where indicated, samples were enriched for large cells by passing through an 11 μm filter prior to staining. Cells were analysed for DNA content using an LSRII flow cytometer on the Indo-1 Violet channel and 10,000 cells were acquired for each sample. Data were analysed using FlowJo v. 10.1r7. Doublets and clumps were excluded using the recommended gating strategy of SSC-H vs SSC-W followed by FSC-H vs. FSC-w, and cells were then gated to exclude auto-fluorescence using unstained pooled haploid and diploid control cells. The gating strategy is provided in S1B Fig. Gates for 1C, 2C, 4C, and 8C were established using H99 (haploid) and KN994B7#16 (diploid) controls incubated in rich media conditions as described above.

### Titan cell proliferation

Cells of each type were pre-grown in YNB and then induced to form Titan cells overnight. After 24 hours, cells were passed through an 11 μm filter and the cells >11 μm were collected, normalized to 10^3^/ml, and then returned to inducing conditions. After 48 hours, cells were collected, fractionated by size (>11 μm, <11 μm), and counted. Titanisation rate was expressed as a ratio of cells <11 μm / >11 μm. Data represent three independent biological replicates. Statistical analyses were performed using Graphpad Prism v7, via unpaired t-test. Variance within the two strains was not statistically significantly different (p = 0.6187).

### Co-culture with *E*. *coli* and *S*. *pneumoniae*

H99 cells were incubated overnight at 30°C, 150 rpm in 5 ml YNB without amino acids + 2% glucose. Cells were inoculated into 1xPBS+ 0.04% glucose (the concentration of glucose present in serum) in the presence or absence of 10^6^ live or heat-killed *E*. *coli* (DH5α) or live *S*. *pneumoniae* (R6). Co-cultures were incubated for 24 hr at 37μC 5%CO_2_ and assessed for Titan formation by microscopy as described above. Co-cultures were not sustainable after 24 without supplementation with additional nutrients, and experiments were therefore terminated. Data representative of triplicate independent experiments are shown.

### Compound identification

To identify compounds of interest, total HI-FCS (Biosera) was loaded onto an AKTA purifier system from GE Healthcare with an Agilent column: Bio SEC-3, 100A, 4.6x300mm at a flow rate of 0.3 ml/min for size exclusion chromatography. For the initial run, 400 μl serum was run in phosphate buffer (25 mM NaH_2_PO_4_, 150 mM NaCl, 0.01% NaN_3_, 2 mM EDTA, pH 7.2), collecting 100 μl fractions in a 96 well plate. The entire plate was screened for capacity to induce Titans by incubated H99 cells pre-grown in YNB+Glucose at OD_600_ = 0.01 in 10% fraction+1xPBS in a 96 well plate format. Plates were examined for Titan cells after 48 and 96 hr. The entire assay was run in triplicate and twice independently using distinct lots of FCS. Inducing fractions were pooled and lyophilized. The residue was resuspended in MeOH and desalted. Then, the solution was submitted to HPLC separations, which were carried out using a Phenomenex reversed-phase (C_18_, 250 × 10 mm, L × i.d.) column connected to an Agilent 1200 series binary pump and monitored using an Agilent photodiode array detector. Detection was carried out at 220, 254, 280 and 350 nm. The entire volume was purified by RP-HPLC using a gradient of MeOH in H_2_O as eluent (50–100% over 70 min, 100% for 20 min) at a flow rate of 1 ml/min. The main fraction was dried, suspended in a minimal volume of DMSO-*d*_*6*_ and submitted to ^1^H-NMR, ^1^H-^13^C HSQC and ^1^H-DOSY analyses. NMR data were acquired on a Bruker 500 MHz spectrometer.

### Ethics statement

All animal experiments were performed under UK Home Office project license PPL 70/9027 which was reviewed and approved by the University of Aberdeen Animal Welfare and Ethical Review Body (AWERB) and the UK Home Office and granted to DMM. Animal experiments adhered to the UK Animals (Scientific Procedures) Act 1986 (ASPA) and European Directive 2010/63/EU on the protection of animals used for scientific purposes. All animal experiments were designed with the 3Rs in mind and were reported using the ARRIVE guidelines.

### Mice and infection models

C57BL/6J mice were bred and maintained in individually ventilated cages (IVCs) at the Medical Research Facility at the University of Aberdeen. Balb/C female mice were obtained from Harlan Laboratories (UK). For each experiment, group size was determined based on previous experiments as the minimum number of mice needed to detect statistical significance (p<0.05) with 90% power (α = 0.05, two-sided). Mice were randomly assigned to groups by an investigator not involved in the analysis and the fungal inocula were randomly allocated to groups. Inocula were delivered in a blinded fashion. Mice were provided with food and water *ad libitum*. Mice were monitored for signs and symptoms of disease. Weight was recorded daily. Mice showing weight loss of greater than 30% (C57BL/6J) or 20% (Balb/C) and signs of disease progression were immediately culled by a schedule one method (cervical dislocation).

C57BL/6J male mice (n = 5/group (immunology) or 10/group (survival), 8–12 weeks old) or Balb/C female mice (n = 10/group, 6–8 weeks old) were anesthetized using injectable anesthesia and infected intranasally with 20 μl PBS suspension containing 10^5^ *C*. *neoformans* (H99 or Zc1) pre-grown in Sabouraud Dextrose medium [8]. For 7 day studies, mice were culled by euthatal injection. Lungs and brains were collected under sterile conditions. Whole brains and one lung lobe were weighed and homogenized for CFU counts. For long term infection studies, mice were observed with daily records of body weights. Mice that reached a predetermined threshold of >25% (C57BL/6J) or >20% (Balb/C) weight loss, or signs and symptoms of neurological disease, were immediately culled. Mice were humanely sacrificed by cervical dislocation following precipitous weight loss (>20%) and the assay was terminated after 28 days. Survival data were assessed by Kaplan Myer and Gehan-Breslow-Wilcoxon test. Lung and brain were sterilely collected, weighed, and homogenized in 1 ml sterile PBS, and 10 μl was plated for CFUs.

### Lung flow cytometry

Lungs from infected mice were used to generate single-cell suspension using mouse lung dissociation kit and the gentleMACS as per manufactures’ instructions (Miltenyi Biotec). Fungal cells were separated from mammalian cells via a 70%/30% discontinuous Percoll gradient centrifugation. Immune cells were stained with the fixable viability dye eFluor 455UV (eBiosecience) for 30 min at 4°C, washed with 1x PBS then fixed with a 2% paraformaldehyde (PFA) solution for 10 min at room temperature. Cell surface staining with antibody cocktail of mAbs specific to CD45-BV650, MHC-II-PECSF594, CD11c-APC, CD11b-BUV395, Ly6C-PE, Ly6G-FITC (all from BD Biosciences) was performed in FACS buffer containing 2% fetal calf serum, 2 mM sodium azide and anti-CD16/32 for 30 min at 4°C, washed then acquired on the BD Fortessa cell analyser (BD Biosciences). FlowJo software v10 (Tree Star) was used for data analysis.

### Statistical analysis of FACS data

Data represent percent live CD45^+^ cells. Statistical analyses were performed using Graph Pad Prism (v 7), and significance was determined using Mann-Whitney U test. Bars represent 95% CI. Variance within the groups was not statistically different (F test to compare variances).

### Histology and *in vivo* Titanisation

For lung histology, C57BL/6J male mice (n = 5/group, 8–12 weeks old) were anesthetized and infected intranasally with 20 μl PBS suspension containing 10^5^ *C*. *neoformans* (H99 or Zc1) pre-grown in Sabouraud Dextrose as above. After 7 days, mice were culled by euthatal injection. Lung sections were preserved in OTC medium and sectioned (2–4 μm) for histology. Fungi were visualized by silver staining and hematoxylin counterstain (Sigma HHS32) using the Sigma-Aldrich Silver Stain modified GMS kit according to the manufacturer’s instructions).

### Bronchoalveolar lavage (BAL)

BAL was performed on male C57BL/6 mice (8–12 weeks) culled by CO_2_ exposure. Lungs were perfused with 1 ml 1xPBS and the collected fluid concentrated by overnight drying on a speedvac. The resulting pellet was weighed, resuspended in sterile PBS, and used at a concentration of 10% w/v in place of FCS in the induction protocol. Animal experiments were performed by ID, ERB, AC, and DMM.

## Supporting information

S1 Fig Characterization of Titan cells. A) H99 was pre-grown in YNB + Glucose overnight at 150 RPM, 30°C and inoculated at OD_600_ = 0.5 and 0.01 into 10% FCS. Representative cells are shown after 7 days. Cells were fixed, permeabilised, and stained (DAPI, 300 ng/ml) to visualize nuclei. Scale bar = 20 μm. Imaged on a Zeiss Axio Observer Z1 at 40x. B) Representative flow cytometry data showing gating strategy to identify cell ploidy. C) Cell body and capsule diameter were measured as indicated for H99 cells induced using the established protocol (n = 299). Capsule width was defined as capsule–cell body diameter. R^2^ values are shown. D) TEM of a Titan cell showing cytoplasm excluded by a large vacuole. E) Control CNV111 cells were quantified for mCherry-Cse4 foci (YPD n = 201, YNB n = 199, p = 0.048).
(TIF)

S2 Fig Flow cytometry showing cell size and DNA content of representative isolates. Individual isolates shown in Fig 3E are further analysed for cell size and DNA content, A) (i) Proportion of cells showing haploid DNA content (gate C1) for each isolate relative to a haploid control (H99); (ii) Size (FSC) and complexity (SSC) of total cell populations for haploid control (H99) and individual isolates. B-D) For each isolate, the DNA content (i) and cell size and complexity (ii) of the total population is shown relative to a haploid control (H99).
(TIF)

S3 Fig Histology of H99 infected mice and serum fractionation by SEC. A) Histology from untreated and Pen/Strep (2,000 U/L) treated H99 infected mice, and resulting lung fungal and bacterial CFUs. B) H99 untreated and Pen/Strep (2000 U/L) treated cells were induced for 24 hr to form Titan cells and degree and size of Titanisation were quantified (n>150; median treated = 7.274±2.855 median untreated 6.286±4.235; p = 0.4248). C) Total HI-FCS was fractionated by size exclusion chromatography. The chromatogram of the total volume is shown.
(TIF)

S4 Fig Titanisation and *in vivo* clinical and environmental isolates. A) Cryptococcus gattii strain R265 was pre-grown in YNB, inoculated into 10%FCS at OD = 0.01, and incubated at 37°C, 5%CO2 for 5 days. Cells were counterstained with India ink to reveal capsule. Scale bar = 10 μm. B) 63 Clinical and environmental isolates were induced for Titan cells (YNB, 10%FCS, OD600 = 0.001) and analysed for increased cell size and cell ploidy (DAPI, flow cytometry). Strains were categorized as Titanising if cells >10 μm were observed, indeterminate if cells >7μm but <10μm were observed, and non-Titanising if only cells < 7um were observed. The percent of strains identified for each category is shown. Representative environmental isolates S8963, Ze14, and Ze18 are shown compared to H99. C) Clinical isolates Zc1, Zc8, and Zc12 were grown in YPD and then spotted on to YPD agar and incubated at 30 or 37°C as indicated. D,E) C57Bl/6J mice (male, 5 per group) were infected intra-nasally with H99 or Zc1 and sacrificed 7 days p.i. and D) the lung fungal burden and percent weight loss recorded. E) Images of representative lungs from infected mice. G) C57Bl/6J mice (male, 10 per group) were infected with H99 or Zc1 intra-nasally and disease severity was monitored for 21 days by weight loss (Mann-Whitney U, p = 0.002). Mice were sacrificed at humane end-point (p = 0.0377) and lung (p = 0.3411) and brain (p<0.0001) fungal burdens were recorded. F) Gating strategy for immune cell recruitment in the lungs of infected mice.
(TIF)

S1 Table Strains used in this study. (PDF)
