# Supplementary figures and images for: The Cryptococcus neoformans Titan cell is an inducible and regulated morphotype underlying pathogenesis
Source: PLoS Pathog. 2018 May 18;14(5):e1006978. doi: 10.1371/journal.ppat.1006978 (PMC5959070; doi:10.1371/journal.ppat.1006978)

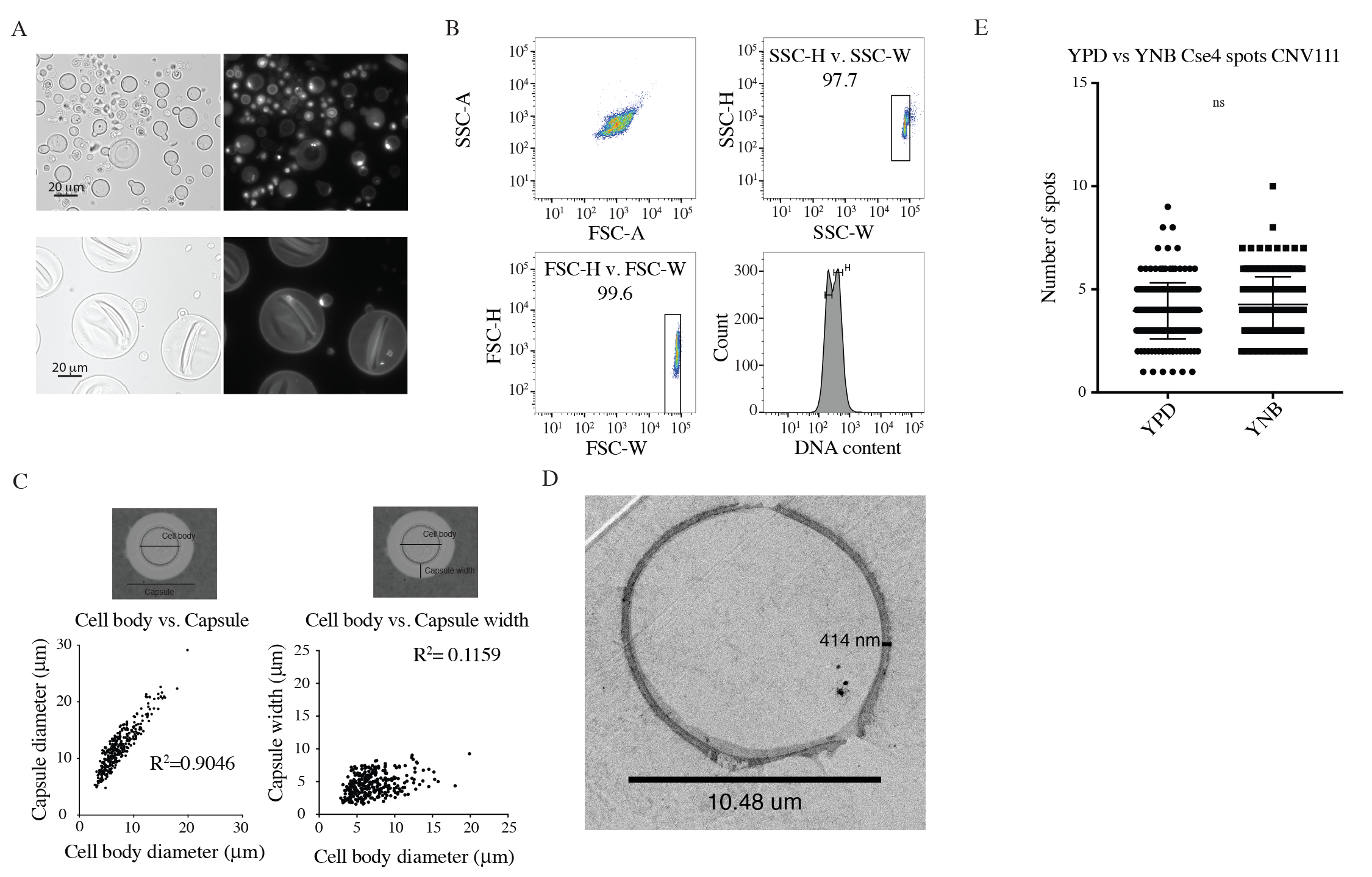

Supplement: S1 Fig — A) H99 was pre-grown in YNB + Glucose overnight at 150 RPM, 30°C and inoculated at OD600 = 0.5 and 0.01 into 10% FCS. Representative cells are shown after 7 days. Cells were fixed, permeabilised, and stained (DAPI, 300 ng/ml) to visualize nuclei. Scale bar = 20 μm. Imaged on a Zeiss Axio Observer Z1 at 40x. B) Representative flow cytometry data showing gating strategy to identify cell ploidy. C) Cell body and capsule diameter were measured as indicated for H99 cells induced using the established protocol (n = 299). Capsule width was defined as capsule–cell body diameter. R2 values are shown. D) TEM of a Titan cell showing cytoplasm excluded by a large vacuole. E) Control CNV111 cells were quantified for mCherry-Cse4 foci (YPD n = 201, YNB n = 199, p = 0.048). (TIF) [file ppat.1006978.s001.tif]

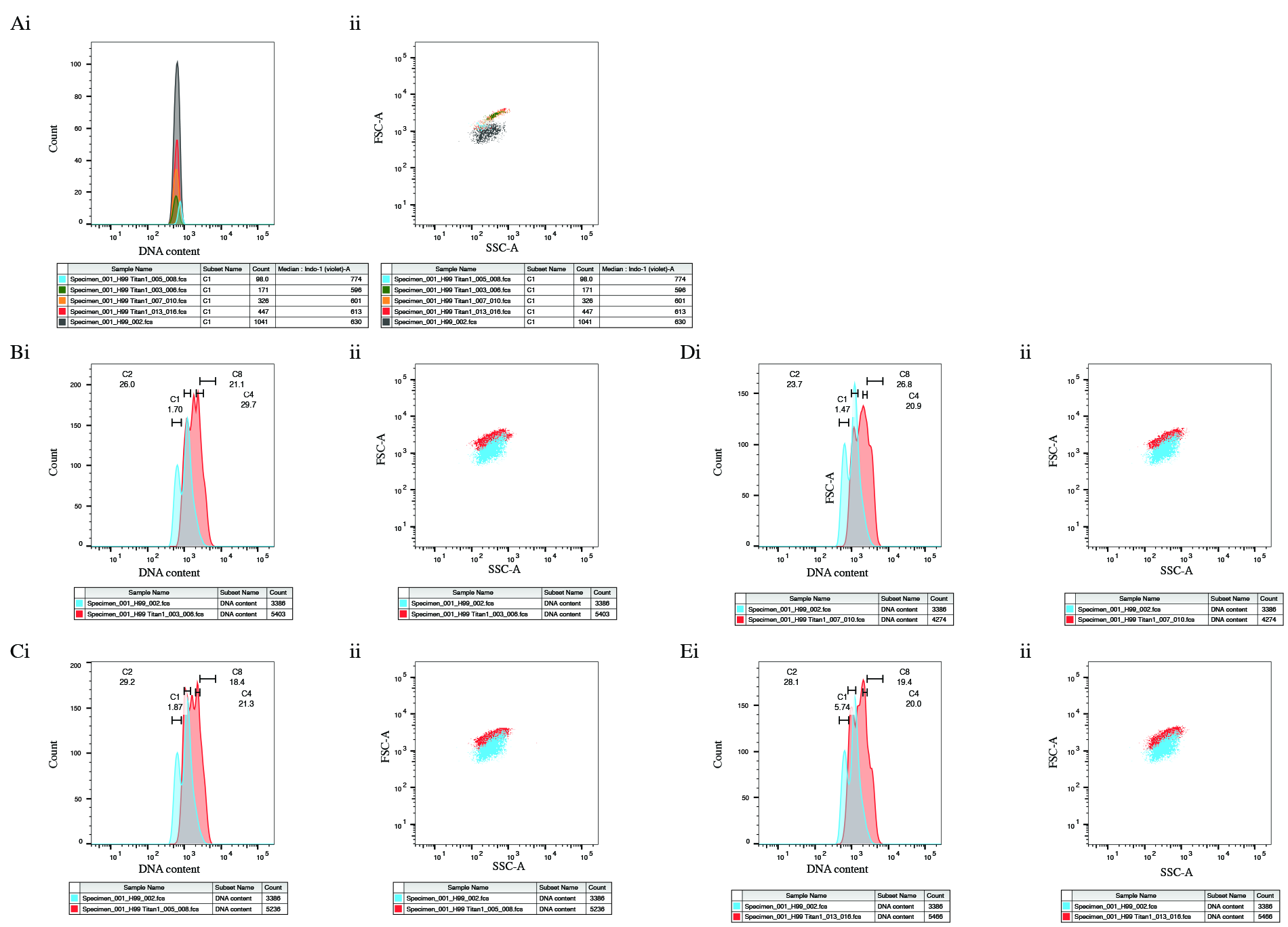

Supplement: S2 Fig — Individual isolates shown in Fig 3E are further analysed for cell size and DNA content, A) (i) Proportion of cells showing haploid DNA content (gate C1) for each isolate relative to a haploid control (H99); (ii) Size (FSC) and complexity (SSC) of total cell populations for haploid control (H99) and individual isolates. B-D) For each isolate, the DNA content (i) and cell size and complexity (ii) of the total population is shown relative to a haploid control (H99). (TIF) [file ppat.1006978.s002.tif]

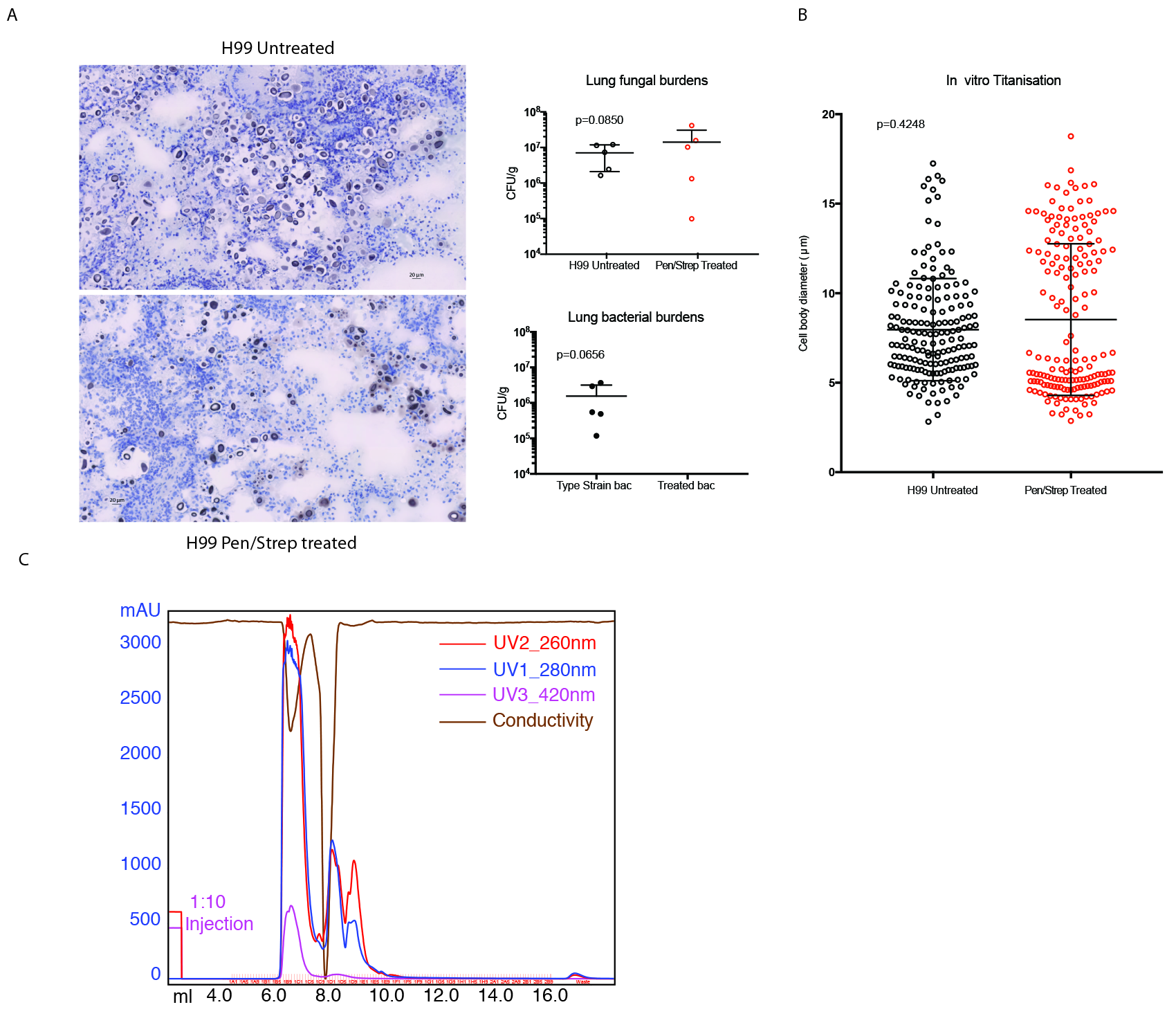

Supplement: S3 Fig — A) Histology from untreated and Pen/Strep (2,000 U/L) treated H99 infected mice, and resulting lung fungal and bacterial CFUs. B) H99 untreated and Pen/Strep (2000 U/L) treated cells were induced for 24 hr to form Titan cells and degree and size of Titanisation were quantified (n>150; median treated = 7.274±2.855 median untreated 6.286±4.235; p = 0.4248). C) Total HI-FCS was fractionated by size exclusion chromatography. The chromatogram of the total volume is shown. (TIF) [file ppat.1006978.s003.tif]

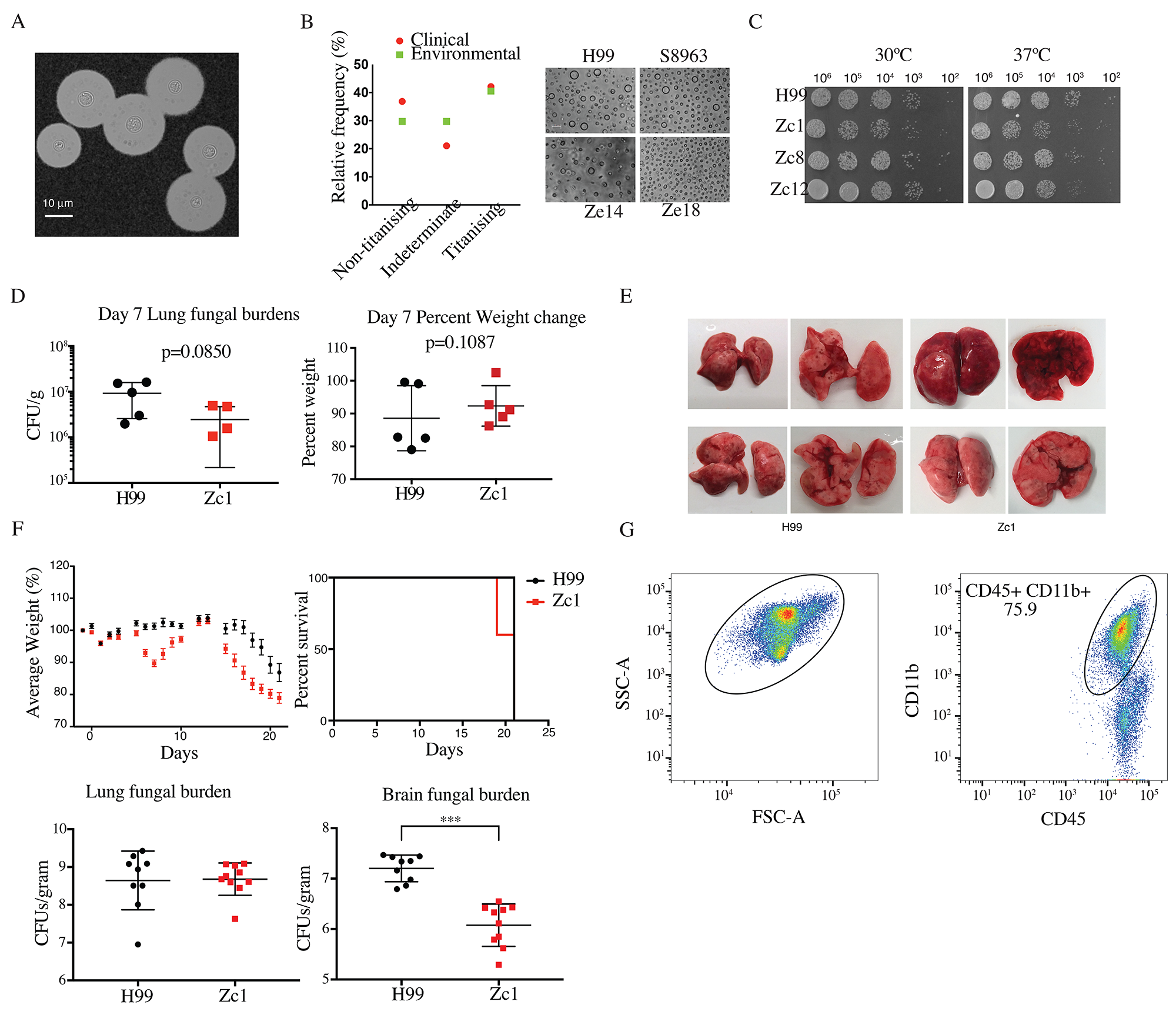

Supplement: S4 Fig — A) Cryptococcus gattii strain R265 was pre-grown in YNB, inoculated into 10%FCS at OD = 0.01, and incubated at 37°C, 5%CO2 for 5 days. Cells were counterstained with India ink to reveal capsule. Scale bar = 10 μm. B) 63 Clinical and environmental isolates were induced for Titan cells (YNB, 10%FCS, OD600 = 0.001) and analysed for increased cell size and cell ploidy (DAPI, flow cytometry). Strains were categorized as Titanising if cells >10 μm were observed, indeterminate if cells >7μm but <10μm were observed, and non-Titanising if only cells < 7um were observed. The percent of strains identified for each category is shown. Representative environmental isolates S8963, Ze14, and Ze18 are shown compared to H99. C) Clinical isolates Zc1, Zc8, and Zc12 were grown in YPD and then spotted on to YPD agar and incubated at 30 or 37°C as indicated. D,E) C57Bl/6J mice (male, 5 per group) were infected intra-nasally with H99 or Zc1 and sacrificed 7 days p.i. and D) the lung fungal burden and percent weight loss recorded. E) Images of representative lungs from infected mice. G) C57Bl/6J mice (male, 10 per group) were infected with H99 or Zc1 intra-nasally and disease severity was monitored for 21 days by weight loss (Mann-Whitney U, p = 0.002). Mice were sacrificed at humane end-point (p = 0.0377) and lung (p = 0.3411) and brain (p<0.0001) fungal burdens were recorded. F) Gating strategy for immune cell recruitment in the lungs of infected mice. (TIF) [file ppat.1006978.s004.tif]
