# Supplementary material for: The Cryptococcus neoformans Titan cell is an inducible and regulated morphotype underlying pathogenesis
Source: PLoS Pathog. 2018 May 18;14(5):e1006978. doi: 10.1371/journal.ppat.1006978 (PMC5959070; doi:10.1371/journal.ppat.1006978)
Supplement: S1 Table — (PDF) [file ppat.1006978.s005.pdf]

**Table S1. *Cryptococcus* strains relevant to this work**				
**Name**	**Description**	**Genotype**	**Parent**	**Source**
H99	Clinical isolate, type strain	VNI MATα		[58]
JKH7	*pka1Δ*	pka1::URA ura- MATα	H99	[59]
CDX19	*gpr4Δgpr5Δ*	*GPR4::NEO GPR5::NAT* MATα	H99	[60]
YSB83	*gpa1Δ*	*GPA1::NAT* MATα	H99	[34]
6E9	*ric8Δ*	*RIC8::NAT* MATα	H99	[62]
19G11	*usv101Δ*	*USV101::NAT* MATα	H99	[62]
YSB42	*cac1Δ*	*CAC1::NAT* MATα	H99	[34]
Zc1	Clinical isolate	VNI MATα		[40]
Zc08	Clinical isolate	VNI MATα		[40]
Zc12	Clinical isolate	VNI MATα		[40]
R265	Clinical isolate, *C. gattii*			[61]
